# A validation of the Japanese adaptation of the Big Five Inventory-2

**DOI:** 10.3389/fpsyg.2022.924351

**Published:** 2022-10-14

**Authors:** Shinya Yoshino, Tadahiro Shimotsukasa, Atsushi Oshio, Yasuhiro Hashimoto, Yuki Ueno, Takahiro Mieda, Ifu Migiwa, Tatsuya Sato, Shizuka Kawamoto, Christopher J. Soto, Oliver P. John

**Affiliations:** ^1^Faculty of Letters, Arts, and Sciences, Waseda University, Tokyo, Japan; ^2^Faculty of Psychology, Rissho University, Tokyo, Japan; ^3^Department of Life Care, Teikyo Junior College, Tokyo, Japan; ^4^Center for Advanced School Education and Evidence-Based Research (CASEER), The University of Tokyo, Tokyo, Japan; ^5^School of Health Sciences, Fukushima Medical University, Fukushima, Japan; ^6^Ministry of Health, Labour and Welfare, Tokyo, Japan; ^7^College of Comprehensive Psychology, Ritsumeikan University, Osaka, Japan; ^8^Faculty of Education, University of Yamanashi, Yamanashi, Japan; ^9^Department of Psychology, Colby College, Waterville, ME, United States; ^10^Department of Psychology, University of California, Berkeley, Berkeley, CA, United States

**Keywords:** Big Five personality, personality measurement, facet, personality structure, BFI, non-WEIRD

## Abstract

The purpose of this study was to adapt a Japanese version of the Big Five Inventory-2 (BFI-2-J) to examine its factor structure, reliability, validity, and measurement invariance. The BFI-2-J assesses five domains and 15 facets of the Big Five personality traits. We analyzed two datasets: 487 Japanese undergraduates and 500 Japanese adults. The results of the principal component analysis and confirmatory factor analysis revealed that the domain-facet structure of the BFI-2-J was similar to that of other language versions. The reliability of the BFI-2-J is sufficient. The correlation coefficients between the BFI-2-J and the other Big Five and self-esteem measures supported convergent and discriminant validity. Moreover, we confirmed measurement invariance across age and sex groups in domain-level and facet-level models. The results suggest that the BFI-2-J is a good instrument for measuring the Big Five personality traits and their facets in Japan. The BFI-2-J is expected to be useful in Japanese personality research and international comparative research.

## Introduction

The Big Five personality traits (or the Five-Factor Model) describe a fundamental and comprehensive framework that classifies various psychological traits into five dimensions: Extraversion, Agreeableness, Conscientiousness, Negative Emotionality (or Neuroticism), and Open-Mindedness (or Openness to Experience). These five dimensions were initially discovered using the lexical approach (e.g., Norman, 1963; Goldberg, 1981; Tupes and Christal, 1992). Subsequent research incorporated constructs from personality questionnaires, leading to the Five-Factor Model (Costa and McCrae, 1992). Therefore, the Big Five and Five-Factor Model differ somewhat in terms of their research methods and detailed interpretation of factors. However, these two models are more alike than different (John et al., 2008). Moreover, the structure of these five factors has been reasonably consistent across cultural areas and countries (McCrae et al., 2005a; Schmitt et al., 2007), although lexical representations of some factors (especially Neuroticism and Openness to Experience) vary across cultures (De Raad et al., 2010), and models with more than five basic traits were developed, such as the HEXACO model (Ashton and Lee, 2007). The Big Five personality traits have been examined not only in research on individual differences in personality but also in research on wider fields such as associations with life outcomes (Ozer and Benet-Martínez, 2006; Roberts et al., 2007; Soto, 2019) and the geographical differences between and within countries (McCrae et al., 2005b; Schmitt et al., 2007; Rentfrow et al., 2008).

The Big Five Inventory (BFI) (John and Srivastava, 1999; John et al., 2008) is one of the most frequently used measures of the lexically based Big Five. The BFI consists of 44 items and assesses each domain with between 8 to 10 items. The BFI is often used because it reflects the meaning of the Big Five personality traits in each item accurately, it is expressed in plain phrases, and consists of useful items (John and Srivastava, 1999; John et al., 2008). Moreover, the BFI has been shown to converge with measures based on trait adjectives, as well as questionnaire representations of the Five-Factor Model (John and Srivastava, 1999; John et al., 2008). The BFI has been translated into other languages and its validity has been confirmed (e.g., Fossati et al., 2011).

The BFI is typically used to assess the scores at the domain level. Soto and John (2017) recently developed the BFI-2 because the Big Five domains are at a high level in the hierarchy of personality structures, and considerable research on facets of the Big Five personality traits has been published since the development of the original BFI (Hofstee et al., 1992; Goldberg, 1993; Saucier and Ostendorf, 1999; DeYoung et al., 2007). Some personality psychology studies have found the importance of examining the characteristics of facets in each domain (Soto and John, 2017). While the Big Five domains summarize a large amount of behavioral information, the facets can predict some specific behaviors more strongly than the domain because of their high fidelity (Paunonen and Ashton, 2001; Soto and John, 2017). Recent studies have also found that the facets of some Big Five domains have distinct patterns on gender (Weisberg et al., 2011) and age trends (Soto et al., 2011). For example, females tend to score higher than males in the sociability and positive affect aspect of Extraversion, whereas males tend to score higher in the assertiveness aspect.

The BFI-2 has demonstrated a robust hierarchical structure at the domain and facet levels and good validity (Soto and John, 2017). Additionally, compared to the BFI, a key advantage of the BFI-2 is that it is easy to control for acquiescent responses (i.e., consistently agreeing or disagreeing with items, regardless of their content) due to the equal number of forward-keyed and reverse-keyed items on the domain and facet scales (Soto and John, 2017). Several types of research using the BFI-2 have already been published, such as relationships with life outcomes (Soto, 2019, 2021). It is possible that BFI-2 may replace the BFI in psychological research in the future.

The original BFI-2 has been translated into German (Danner et al., 2016; Rammstedt et al., 2020), Dutch (Denissen et al., 2020), Slovak (Halama et al., 2020), Russian (Shchebetenko et al., 2020), Danish (Vedel et al., 2021), Chinese (Zhang et al., 2021), Turkish (Cemalcilar et al., 2021), and Norwegian (Føllesdal and Soto, 2022). The development of many language scales of BFI-2 would encourage comparison not only at the domain level (McCrae et al., 2005b; Schmitt et al., 2007) but also at the facet level among cultural areas or countries. Moreover, a growing body of recent research in psychology has focused on non-WEIRD (Western, educated, industrialized, rich, and democratic) populations (Henrich et al., 2010). Previous studies have found associations between life outcomes and psychological variables with the Big Five personality traits (Ozer and Benet-Martínez, 2006; Roberts et al., 2007; Soto, 2019). However, these results were usually observed in Western populations, and it was unclear whether they were replicated in the non-WEIRD population. Cemalcilar et al. (2021) examined the predictive validity of BFI-2 in the Turkish population in a non-WEIRD area. They found that many trait-outcome associations replicated the results of Ozer and Benet-Martínez (2006) and Soto (2019), as well as some cultural differences involving religiosity and social dominance. Evidence of similarities and differences in psychological associations will promote understanding across cultural contexts. It is necessary to translate the BFI-2 into various languages to examine the generalizability of the Big Five personality traits and their predictive validity across cultural areas and countries.

Research on personality structures and the Big Five personality traits has also been conducted in Japan, with a major focus on the lexical approach. Some personality terms have been identified in Japanese dictionaries (Aoki, 1971; Tsuji, 2001; Murakami, 2002; Hashimoto, 2018). Based on these Japanese trait adjectives, previous studies have examined the taxonomy of personality traits and replicated five dimensions as a comprehensive personality structure (Murakami, 2003; Kashiwagi et al., 2005).

There are several Japanese Big Five measures. Some scales have been translations of English measures, such as the NEO-PI-R (Costa and McCrae, 1992; Shimonaka et al., 1999) and the Ten-Item Personality Inventory (TIPI) (Gosling et al., 2003; Oshio et al., 2012, 2013, 2014), while others were developed as original Japanese measures, such as the Big Five Scale based on trait adjectives (BFS) (Wada, 1996), the Five-Factor Personality Questionnaire (FFPQ) (Study Group of the FFPQ, 1998), and the Big Five Personality Inventory (BFPI) (Murakami and Murakami, 1999). The BFI-2 has some merits in compensating for the shortcomings of the existing Japanese Big Five measures. First, BFI-2 items are a reasonably large pool of clear, descriptive phrases. The BFS (Wada, 1996) is a major Big Five measure in Japan. The number of citations of the BFS’s report was 150 as of July 10, 2022 on Google Scholar, while the NEO-PI-R’s report (Shimonaka et al., 1999) was 24, the FFPQ-50 (Fujishima et al., 2005) was 30, and the BFPI (Murakami and Murakami, 1999) was 44. However, the BFS’s items are single-word adjectives that can be ambiguous to interpret (John et al., 2008). The BFI and BFI-2 have been considered as measures consisting of simple and plain phrases compared with the scales of trait adjectives (John et al., 2008; Soto and John, 2017). Moreover, the TIPI-J (Oshio et al., 2012) and the short form of the BFS (BFS-S; Namikawa et al., 2012), which are short instruments, are currently widely used. Although these very brief measures are suited to surveys that include many items to reduce the burden on respondents, the constructs of personality traits covered by them have a narrow range and are sometimes unclear (Sleep et al., 2021). By contrast, the BFI-2 can assess both domains and facets using a sufficient number of items and has a robust structure. Second, the BFI-2 matches international comparative research because it has been translated into various languages. The original Japanese measures—namely the FFPQ and the BFPI—have not been translated into other languages, and are therefore not suitable for cross-cultural research. Additionally, there is no published research on the development of the Japanese version of the BFI, although studies using this measure in Japan have been reported (e.g., Schmitt et al., 2007). We therefore believe that evidence regarding the adaptation of a Japanese-language BFI-2 would contribute to the growth of personality research in Japan.

For these reasons, the purpose of this study was to adapt a Japanese version of the BFI-2 (BFI-2-J) using both student and community samples. First, we investigated the hierarchical factor structure of the BFI-2-J using principal component analysis (PCA) and confirmatory factor analysis. The BFI-2-J assesses each Big Five domain using 12 items and each facet using four items as in the BFI-2. Extraversion includes facets of Sociability, Assertiveness, and Energy Level. Agreeableness includes the facets of Compassion, Respectfulness, and Trust. Conscientiousness includes the facets of Organization, Productiveness, and Responsibility. Negative Emotionality includes facets of Anxiety, Depression, and Emotional Volatility. Open-Mindedness includes the facets of Intellectual Curiosity, Aesthetic Sensitivity, and Creative Imagination. Second, we examined reliability and validity by confirming the test–retest correlations, internal consistency indices, and convergent and discriminant validity of the BFI-2-J using other Big Five measures, as well as self-esteem. Previous research indicates that self-esteem is strongly, positively associated with Extraversion and negatively associated with Negative Emotionality (Robins et al., 2001; Halama et al., 2020). There is also a positive association between self-esteem and Conscientiousness, but Agreeableness and Open-Mindedness are not related to it when compared with these three domains (Robins et al., 2001). These associations have proven to be robust across cultural areas (Gebauer et al., 2015; Vazsonyi et al., 2015). Third, we investigated the measurement invariance of the BFI-2-J across age and sex groups. In research on the Russian adaptation, Shchebetenko et al. (2020) examined the measurement invariance of the BFI-2 across sex and age groups (25 years and younger versus 26 years and older). The result supported strict measurement invariance of the BFI-2’s hierarchical structure across both groups. The BFI-2-J is expected to be useful in wide participants by examining the BFI-2-J’s measurement invariance across demographic groups. To do this, we conducted a multigroup analysis of the domain-level and facet-level structures within each domain.

## Materials and methods

### Participants and procedures

This study consisted of a student sample and a community sample. The participants in the student sample included 495 undergraduate students at Waseda University in Tokyo and Ritsumeikan University in Kyoto and Osaka. The participants in Waseda University in Tokyo were 330 students responding to questionnaires after class on April 18, 2018 (Time 1) and 252 students on May 9, 2018 (Time 2). For students who participated at both times, we used the responses at Time 1. Participants at Ritsumeikan University in Kyoto and Osaka were recruited from the International Situations Project (Lee et al., 2020). One hundred students responded to an online questionnaire, including the BFI-2-J. Lee et al. (2020) did not examine the personality structure in Japan and just scored the Big Five domains in each country. Omitting four non-Japanese undergraduate students and four responses with many missing values (i.e., above 30 items) on the BFI-2-J yielded a final sample of 487 undergraduate students (287 females, 186 males, and 14 who did not report gender; mean age = 20.01, *SD* = 3.92). Most (95%) were under the age of 25 years.

We examined reliability and validity using the participants from Waseda University in Tokyo. A total of 171 undergraduate students participated at both Time 1 and Time 2, which was used to examine the test–retest reliability. Moreover, at each time point, two questionnaire forms were administered. There were thus a total of four forms: one including the BFS-S and the TIPI-J; one including the FFPQ-50; one including the BFPI; and one including the NEO-FFI. The former two questionnaires were distributed randomly at Time 1, and the latter two questionnaires were distributed randomly at Time 2. All four forms also included the BFI-2-J, and were therefore to examine convergent and discriminant validity. The number of responses to the BFS-S and TIPI-J, FFPQ-50, BFPI, and NEO-FFI was 150, 152, 87, and 119, respectively. The sample size for the BFPI was smaller than others due to researcher error. However, this sample size is still large enough to detect convergent correlations (e.g., 95% power to detect correlations stronger than 0.36).

The participants in the community sample included 500 Japanese adults (250 females and 250 males; mean age = 44.94, *SD* = 14.07). We conducted an online survey in April 2019 with Agekke Corporation^1^ to recruit a sample that was more diverse in terms of age (with 100 adults in their 20s, 30s, 40s, 50s, and 60s, respectively) to examine measurement invariance across age and sex groups. Two hundred thirty-seven participants had university degree or more; 83 participants had completed vocational school; 150 participants had completed junior high school, 17 had completed high school; and 13 participants did not report educational level. These participants responded to an online questionnaire, including the BFI-2-J and a self-esteem measure, to examine predictive validity.

### Measures

We used seven scales, including six Big Five measures and one self-esteem measure. The BFI-2-J was completed by all participants. Other Big Five measures were completed by a part of the student sample, as described above, and the self-esteem measure was completed by the community sample. Table 1 presents the measures included in each survey.

**Table 1:** Survey and assessing measures.

				Big Five measure						Self esteem
				BFI-2-J	TIPI-J	BFS-S	FFPQ-50	BFPI	NEO-FFI	RSES-J
Student										
	Kyoto and Osaka			ₒ						
										
	Tokyo									
		Time 1								
			Ver. 1	ₒ	ₒ	ₒ				
			Ver. 2	ₒ			ₒ			
		Time 2								
			Ver. 1	ₒ				ₒ		
			Ver. 2	ₒ					ₒ	
Community				ₒ						ₒ

#### Japanese version of Big Five Inventory

The BFI-2 (Soto and John, 2017) was translated from English into Japanese after obtaining the authors’ permission. We translated fluent Japanese text that retained each item’s original meaning, and considered multiple translation options for some items. In a series of pilot studies, we removed items with poor convergent correlations or low principal component loadings, and wrote new items to replace them. The final set of 60 items was decided after seven pilot studies, which were participated in by 30, 21, 53, 73, 67, 72, and 227 undergraduates, respectively. The BFI-2-J was then re-translated into English by a native English speaker at a translation and editing service. The authors of the English-language BFI-2 checked for discrepancies from the original version. We then slightly revised the language used in some items.

The final BFI-2-J was included in all the surveys. The BFI-2-J consists of 60 items, with 12 items assessing each domain and four items assessing each facet. Each item was rated on a 5-point scale ranging from 1 (strongly disagree) to 5 (strongly agree). The Japanese version of the items is presented in the Supplementary Table S1.

#### Short form of the Big Five Scale (Japanese original)

The BFS-S (Namikawa et al., 2012) consists of 29 Japanese trait adjectives and assesses each domain with five to seven items. This scale was developed by using item response theory to select a subset of items from the full BFS, which consists of 60 trait adjectives (Wada, 1996). Each item was rated on a 7-point scale ranging from 1 (strongly disagree) to 7 (strongly agree). In the sample of this survey, Cronbach’s alpha coefficients were 0.89, 0.80, 0.82, 0.88, and 0.79 for Extraversion, Agreeableness, Conscientiousness, Negative Emotionality, and Open-Mindedness, respectively.

#### Japanese version of the Ten-Item Personality Inventory

The TIPI-J (Oshio et al., 2012, 2013, 2014) consists of 10 items translated from the TIPI (Gosling et al., 2003) and assesses each domain with two items. Each item was rated on a 7-point scale ranging from 1 (strongly disagree) to 7 (strongly agree). The previous study also examined the validity across languages (Oshio et al., 2014). In the present sample, correlation coefficients between the two items measuring each Big Five domain were 0.64, 0.30, 0.26, 0.40, and 0.45 for Extraversion, Agreeableness, Conscientiousness, Negative Emotionality, and Open-Mindedness, respectively.

#### Five-Factor Personality Questionnaire-50 (Japanese original)

The FFPQ-50 (Fujishima et al., 2005) consists of 50 items that assess each Big Five domain with 10 items. The FFPQ-50 is a shortened version of the original, 150-item FFPQ (Study Group of the FFPQ, 1998). Each item was rated on a 5-point scale ranging from 1 (strongly disagree) to 5 (strongly agree). In the present sample, Cronbach’s alpha coefficients were 0.81, 0.80, 0.82, 0.87, and 0.77 for Extraversion, Agreeableness, Conscientiousness, Negative Emotionality, and Open-Mindedness, respectively.

#### Big Five Personality Inventory (Japanese original)

The BFPI (Murakami and Murakami, 1999) consists of 12 items assessing each Big Five domain. Each item was responded to with “Yes” (coded 1) or “No” (coded 0). In the present sample, Cronbach’s alpha coefficients were 0.91, 0.82, 0.82, 0.90, and 0.75 for Extraversion, Agreeableness, Conscientiousness, Negative Emotionality, and Open-Mindedness, respectively.

#### Japanese version of NEO Five-Factor Inventory

The NEO-FFI (Shimonaka et al., 1999) consists of 60 items translated from the original, English-language version (Costa and McCrae, 1992) and assesses each Big Five domain with 12 items. Each item was rated on a 4-point scale ranging from 1 (strongly disagree) to 4 (strongly agree). In the present sample, Cronbach’s alpha coefficients were 0.86, 0.74, 0.75, 0.78, and 0.69 for Extraversion, Agreeableness, Conscientiousness, Negative Emotionality, and Open-Mindedness, respectively.

#### Japanese version of Rosenberg’s self-esteem scale

The RSES-J (Sakurai, 2000) consists of 10 items translated from Rosenberg’s self-esteem scale (RSES) (Rosenberg, 1965). Each item was rated on a 4-point scale ranging from 1 (strongly disagree) to 4 (strongly agree). In the present sample, Cronbach’s alpha coefficient was 0.86.

### Ethical concerns

This study was approved by the Research Ethics Committee of Waseda University (application number 2017-HN026). The participants were informed of the following: participation in the survey was voluntary, no disadvantage would be caused by participation or non-participation, participants could stop answering at any time during the survey, and responses would be collected anonymously. They took part in the survey after consenting to participate.

### Analysis plan

Our analysis closely follows the plan of Soto and John (2017), in order to directly compare the results obtained for the BFI-2-J with the English-language source version of the BFI-2. First, we examined the factor structure of each student and community sample. We confirmed the basic domain-level structure using 60 items with a PCA after centering each individual’s set of item responses around their within-person mean to control for acquiescence at the item level (Soto and John, 2017). Additionally, we conducted a PCA using the means of the 15 facet scales. Next, we examined the facet-level structure within each Big Five domain using a confirmatory factor analysis (CFA). Second, we investigated the reliability and validity of the BFI-2-J. We examined the test–retest reliability by asking the participants to take part both times in the student sample. There was an interval of 3 weeks between times 1 and 2. Internal consistency was assessed using Cronbach’s alpha and Omega coefficients for both samples. We also examined convergent and discriminant validity using the correlation coefficients between the BFI-2-J and the other Big Five measures in the student sample. Moreover, we analyzed the correlation coefficients between the BFI-2-J and self-esteem measure in the community sample. Third, we examined measurement invariance (MI) across age and sex groups in the domain-level structure and facet-level structures within each domain using the dataset of the community sample. The responses were divided into age groups of <45 (*n* = 239) and ≥45 years (*n* = 261) because their median age was 45.50. According to the most recent national census, the Japanese median age is 48.61;^2^ thus, a cutoff of 45 years closely reflects the status of classifying the Japanese population as younger or older people. We compared the fit of the models by imposing restrictions at the four levels of invariance: configural, metric, strong, and strict. Configural invariance includes the same items and number of factors for each group. Next, metric invariance indicates that factor loading parameters are equal across groups, which are added to configural invariance. In addition to metric invariance, strong invariance means that the item intercepts are invariant across groups. Finally, strict invariance also constrains the residual variances to be equal across groups.

We used R (version 3.6.2; R Core Team, 2019) to analyze the datasets.

## Results

### Factor structure

We examined the domain-level structure of the BFI-2-J in the student and community samples. We used 60 items after computing each participant’s mean response across the full set of 60 items (prior to reverse-coding the negatively keyed items), and then subtracting this within-person mean from each of their individual item responses. In each sample, visual inspection of the pattern of eigenvalues suggested five components above the scree line (Supplementary Figures S1, S2). Moreover, the parallel analysis suggested the extraction of five components in the community sample and six components in the student sample. Table 2 presents the results of the PCA extracted and varimax rotated five components. Items usually had absolute principal loadings ranging from 0.30 or stronger on the intended component, although there were several cross-loadings. Averaged across the 60 BFI-2-J items, the mean absolute values of the intended primary loadings were 0.59 (student sample) and 0.56 (community sample), whereas the mean absolute values of the remaining loadings were only 0.11 (student sample) and 0.15 (community sample). Additionally, to test the robustness of these results, we also examined the domain-level structure using a PCA with oblimin rotation, an exploratory factor analysis (EFA; maximum likelihood method) with varimax rotation, and an EFA with oblimin rotation (Supplementary Tables S2–S4). Across the four analyses (PCA or EFA extraction × varimax or oblimin rotation), correlations between corresponding pairs of factor or component scores averaged 96 in the student sample and 0.88 in the community sample. These results indicate that the BFI-2-J’s domain-level structure was robust across variations in extraction and rotation.

**Table 2:** Loadings from a principal components analysis of the 60 items.

		Student					Community				
	Items	E	A	C	N	O	E	A	C	N	O
**Extraversion**											
Sociability											
	Item 01	**0.76**	0.20	−0.01	0.19	0.05	**0.70**	0.23	0.17	**0.32**	0.04
	Item 16	**−0.76**	−0.08	0.09	0.01	−0.06	**−0.73**	−0.16	0.00	0.04	−0.07
	Item 31	**−0.81**	0.00	−0.06	−0.12	−0.06	**−0.72**	−0.08	−0.21	−0.29	−0.03
	Item 46	**0.67**	0.01	−0.09	0.04	−0.03	**0.69**	0.12	−0.13	0.04	−0.05
Assertiveness											
	Item 06	**0.80**	0.05	0.04	0.14	0.11	**0.67**	0.12	0.20	**0.31**	0.08
	Item 21	**0.66**	−0.08	0.22	0.07	0.05	**0.45**	−0.04	**0.43**	0.22	0.10
	Item 36	**−0.40**	0.21	−0.20	−0.19	−0.19	**−0.35**	0.08	−0.25	−0.18	−0.26
	Item 51	**−0.58**	0.16	−0.28	−0.01	−0.12	**−0.46**	0.20	**−0.40**	−0.10	−0.19
Energy level											
	Item 11	**−0.49**	−0.09	0.28	0.21	−0.11	**−0.51**	0.15	0.19	0.25	−0.12
	Item 26	**−0.78**	−0.12	−0.12	−0.07	−0.12	**−0.67**	−0.08	**−0.32**	**−0.31**	−0.09
	Item 41	**0.68**	0.19	0.10	0.18	0.06	**0.53**	0.10	0.24	**0.41**	0.17
	Item 56	**0.61**	0.16	−0.16	−0.12	0.07	**0.58**	0.03	−0.01	0.17	0.14
**Agreeableness**											
Compassion											
	Item 02	0.18	**0.56**	0.15	0.18	0.13	0.22	**0.61**	0.27	0.06	0.11
	Item 17	−0.27	**−0.38**	−0.03	0.26	−0.23	−0.26	**−0.49**	−0.06	0.19	−0.23
	Item 32	0.02	**0.49**	0.16	0.03	0.03	0.17	**0.39**	0.08	0.18	0.10
	Item 47	−0.17	**−0.62**	−0.05	−0.05	0.03	−0.27	**−0.57**	−0.18	−0.02	−0.20
Respectfulness											
	Item 07	0.05	**0.58**	0.21	0.01	0.09	0.14	**0.63**	0.19	0.02	0.12
	Item 22	**0.31**	**−0.43**	−0.08	**−0.31**	−0.01	0.22	**−0.52**	−0.09	−0.06	0.09
	Item 37	−0.03	**−0.49**	−0.10	−0.23	0.15	0.03	**−0.59**	−0.15	−0.23	−0.06
	Item 52	−0.06	**0.58**	0.16	−0.02	0.05	0.03	**0.62**	**0.34**	−0.02	0.10
Trust											
	Item 12	0.10	**−0.36**	0.12	**−0.33**	−0.01	0.05	**−0.40**	0.08	**−0.36**	−0.01
	Item 27	−0.07	**0.42**	−0.02	**0.40**	0.05	−0.14	**0.53**	−0.06	0.29	0.12
	Item 42	−0.15	**−0.37**	−0.05	**−0.35**	0.04	−0.07	**−0.37**	0.08	**−0.45**	−0.13
	Item 57	0.23	**0.44**	−0.12	0.20	0.11	0.23	**0.39**	0.12	0.24	0.13
**Conscientiousness**											
Organization											
	Item 03	0.17	−0.06	**−0.62**	0.09	0.03	0.04	−0.06	**−0.61**	0.09	−0.03
	Item 18	−0.18	−0.05	**0.63**	0.02	0.01	−0.09	0.10	**0.66**	−0.07	0.03
	Item 33	−0.07	0.02	**0.62**	−0.01	0.11	0.00	0.07	**0.61**	0.01	0.11
	Item 48	0.01	−0.11	**−0.60**	−0.02	−0.10	−0.03	−0.19	**−0.62**	0.00	−0.08
Productiveness											
	Item 08	−0.16	−0.12	**−0.50**	0.09	−0.11	−0.27	−0.04	**−0.58**	−0.10	−0.06
	Item 23	−0.21	−0.06	**−0.57**	−0.09	0.03	−0.14	−0.17	**−0.55**	−0.25	0.06
	Item 38	0.19	0.05	**0.55**	0.19	−0.06	0.18	0.15	**0.58**	0.15	0.16
	Item 53	0.09	**0.38**	**0.40**	0.09	−0.11	0.08	**0.37**	**0.53**	0.09	0.04
Responsibility											
	Item 13	0.01	0.17	**0.65**	−0.07	−0.04	0.12	**0.34**	**0.52**	−0.07	0.02
	Item 28	0.02	0.04	**−0.55**	−0.03	0.12	0.00	−0.03	**−0.59**	−0.21	0.02
	Item 43	**0.33**	0.13	**0.57**	0.05	−0.05	**0.31**	0.17	**0.48**	0.13	0.12
	Item 58	0.00	−0.20	**−0.56**	0.01	0.10	−0.11	**−0.36**	**−0.53**	−0.12	−0.12
**Negative emotionality**											
Anxiety											
	Item 04	0.06	0.13	−0.02	**0.76**	−0.03	0.22	0.10	0.17	**0.70**	0.14
	Item 19	−0.17	−0.04	0.18	**−0.62**	0.05	−0.05	0.03	0.07	**−0.68**	0.06
	Item 34	−0.14	−0.04	0.03	**−0.75**	0.05	−0.23	−0.07	−0.09	**−0.73**	−0.03
	Item 49	0.10	−0.10	0.02	**0.61**	−0.19	0.14	−0.09	0.10	**0.73**	0.00
Depression											
	Item 09	0.06	−0.10	−0.29	**0.54**	0.06	0.16	−0.03	−0.14	**0.64**	0.10
	Item 24	0.15	0.23	−0.03	**0.58**	−0.02	0.20	0.22	0.08	**0.60**	0.16
	Item 39	−0.17	−0.05	−0.05	**−0.72**	0.13	−0.21	−0.11	−0.18	**−0.69**	0.03
	Item 54	−0.27	−0.02	−0.10	**−0.73**	0.12	−0.29	−0.11	−0.26	**−0.69**	−0.05
Emotional volatility											
	Item 14	0.10	−0.23	−0.19	**−0.59**	−0.05	0.01	**−0.45**	−0.25	**−0.48**	−0.08
	Item 29	−0.11	0.05	0.22	**0.73**	−0.08	−0.03	0.26	**0.35**	**0.59**	0.00
	Item 44	−0.09	0.25	0.27	**0.60**	−0.03	−0.08	**0.35**	**0.38**	**0.42**	0.02
	Item 59	0.08	−0.21	−0.06	**−0.69**	−0.06	0.05	**−0.31**	−0.26	**−0.57**	−0.15
**Open-mindedness**											
Intellectual curiosity											
	Item 10	**0.38**	0.06	0.02	0.08	**0.49**	**0.34**	0.09	0.25	0.19	**0.36**
	Item 25	0.00	−0.03	−0.11	0.19	**−0.48**	−0.12	0.05	−0.23	0.14	**−0.33**
	Item 40	−0.01	−0.01	0.18	**−0.44**	**0.34**	−0.06	−0.12	0.20	**−0.45**	0.23
	Item 55	−0.05	0.03	0.00	0.12	**−0.55**	−0.07	−0.18	−0.12	−0.07	**−0.54**
Aesthetic sensitivity											
	Item 05	0.07	−0.08	0.08	0.13	**−0.77**	−0.01	−0.12	0.02	0.02	**−0.75**
	Item 20	−0.09	0.09	−0.09	−0.07	**0.69**	0.01	0.16	−0.07	0.00	**0.80**
	Item 35	−0.08	0.04	−0.07	−0.11	**0.71**	−0.05	0.14	−0.05	0.04	**0.74**
	Item 50	−0.08	−0.18	0.04	0.13	**−0.56**	−0.13	**−0.31**	0.01	0.09	**−0.60**
Creative imagination											
	Item 15	0.20	−0.12	0.04	0.13	**0.61**	0.26	−0.09	**0.47**	0.29	**0.40**
	Item 30	−0.28	0.05	−0.04	−0.05	**−0.62**	−0.26	−0.03	**−0.40**	−0.16	**−0.56**
	Item 45	−0.13	−0.12	0.13	−0.04	−0.23	−0.23	−0.11	−0.21	−0.19	**−0.40**
	Item 60	**0.31**	−0.14	−0.01	0.08	**0.63**	**0.31**	−0.16	**0.31**	0.21	**0.51**

Each individual item’s response was subtracted from the within-person mean. Absolute loadings of 0.30 or stronger are bolded. The participants in the student and community sample included 487 and 500 Japanese adults, respectively. Cumulative proportion of variance is 0.44 (student sample) and 0.46 (community sample).

We also examined the domain-level structure using the mean of the raw item scores for each facet. In the PCA extracted and varimax rotated five components, all 15 facets had the strongest loading on the intended component in both samples (Table 3). All these primary loadings were at least 0.62, and averaged 0.79 (student sample) and 0.77 (community sample). Taken together, these results indicate that the BFI-2-J’s intended Big Five structure can be clearly recovered from both its items and facet scales.

**Table 3:** Loadings from a principal components analysis of the 15 facet scores.

	Student					Community				
	E	A	C	N	O	E	A	C	N	O
**Extraversion**										
Sociability	**0.87**	0.08	−0.02	−0.11	0.05	**0.86**	0.18	0.06	−0.15	0.03
Assertiveness	**0.80**	−0.10	0.24	−0.14	0.14	**0.70**	−0.03	0.35	−0.25	0.23
Energy level	**0.86**	0.14	−0.04	0.01	0.13	**0.84**	0.05	0.07	−0.18	0.16
**Agreeableness**										
Compassion	0.25	**0.78**	0.14	0.05	0.12	0.32	**0.76**	0.22	0.01	0.21
Respectfulness	−0.15	**0.76**	0.22	−0.20	−0.05	−0.05	**0.80**	0.33	−0.12	0.07
Trust	0.06	**0.62**	−0.05	−0.48	0.05	0.05	**0.69**	−0.04	−0.50	0.06
**Conscientiousness**										
Organization	−0.17	0.03	**0.81**	0.02	0.06	−0.01	0.08	**0.86**	0.01	0.13
Productiveness	0.22	0.17	**0.76**	−0.10	0.01	0.27	0.18	**0.75**	−0.23	0.09
Responsibility	0.10	0.11	**0.85**	0.01	−0.09	0.22	0.28	**0.75**	−0.16	0.09
**Negative emotionality**										
Anxiety	−0.15	−0.05	0.05	**0.89**	0.12	−0.26	−0.02	−0.06	**0.89**	0.01
Depression	−0.21	−0.06	0.05	**0.87**	0.09	−0.33	−0.13	−0.10	**0.85**	−0.03
Emotional volatility	0.13	−0.22	−0.22	**0.78**	0.03	0.02	−0.37	−0.37	**0.67**	−0.09
**Open-mindedness**										
Intellectual curiosity	0.13	0.00	0.11	0.22	**0.75**	0.17	0.01	0.26	0.08	**0.73**
Aesthetic sensitivity	−0.07	0.11	−0.09	0.11	**0.80**	0.01	0.26	−0.09	−0.04	**0.81**
Creative imagination	0.32	−0.04	−0.04	−0.14	**0.71**	0.38	0.00	0.35	−0.30	**0.62**

Facet scores were calculated using the average of four items in each facet. Absolute loadings of 0.60 or stronger are bolded. The participants in the student and community sample included 487 and 500 Japanese adults, respectively. Cumulative proportion of variance is 0.72 (student sample) and 0.76 (community sample).

At the end of this section, we examined facet-level structures within each Big Five domain using CFA (maximum likelihood method). Following the statistical procedures of previous BFI-2 research (Soto and John, 2017; Halama et al., 2020; Vedel et al., 2021), we compared five models fit to the raw item scores within each domain: single domain model (a single factor defined all 12 items within a domain), single domain plus acquiescence model (the substantive domain factor and an acquiescence method factor), forward-keyed and reverse-keyed items model (two correlated factors defined by the domain’s forward-keyed and reverse-keyed items), three facets model (three factors representing the three BFI-2 facet scales within a domain), and three facets plus acquiescence model (three factors representing the three BFI-2 facet scales and an acquiescence method factor). We report the Chi-square, comparative fit index (CFI), Tucker–Lewis index (TLI), root mean square error of approximation (RMSEA), and Bayesian information criterion (BIC) as fit statistics for these models in Table 4. Based on conventional guidelines (e.g., Marsh et al., 2004), we interpreted values greater than 0.90 for CFI and TLI and smaller than 0.08 for RMSEA as indicating acceptable model fit. In both samples, the three facets plus acquiescence model provided an acceptable fit for Conscientiousness, Negative Emotionality, and Open-Mindedness, except for TLI of Conscientiousness in the community sample, although this model did not have a good fit for Extraversion and Agreeableness. In terms of Extraversion, the goodness of fit of the three facets plus acquiescence model was improved by including error covariance between items 16 and 46 for both samples (student sample: *χ^2^* (49) = 309.11, CFI = 0.91, TLI = 0.88, RMSEA = 0.10, BIC = 14849.52; community sample: *χ^2^* (49) = 258.55, CFI = 0.92, TLI = 0.89, RMSEA = 0.09, BIC = 15588.42). The fit statistics of structure within Agreeableness did not improve by including *ad hoc* error co-variances. The factor loadings in the three facets plus acquiescence model are shown in the Supplementary Table S5. These results indicate that the BFI-2’s intended facet-level structure can be clearly recovered for four of the five Big Five domains.

**Table 4:** Fit statistics for confirmatory factor analyses of BFI-2-J items.

	Student						Community					
	*χ^2^*	*df*	CFI	TLI	RMSEA	BIC	*χ^2^*	*df*	CFI	TLI	RMSEA	BIC
**Extraversion**												
Single domain	495.86	54	0.85	0.81	0.13	15005.32	460.77	54	0.84	0.80	0.12	15759.57
Single domain plus acquiescence	489.21	53	0.85	0.81	0.13	15004.86	412.47	53	0.86	0.82	0.12	15717.48
Forward and reverse keyed items	482.53	53	0.85	0.81	0.13	14998.19	404.46	53	0.86	0.82	0.12	15709.48
Three facets	447.19	51	0.86	0.82	0.13	14975.22	433.68	51	0.85	0.80	0.12	15751.12
Three facets plus acquiescence	431.90	50	0.87	0.82	0.13	14966.12	362.60	50	0.87	0.83	0.11	15686.26
**Agreeableness**												
Single domain	441.40	54	0.62	0.54	0.12	15287.86	540.14	54	0.67	0.60	0.13	15024.47
Single domain plus acquiescence	352.65	53	0.71	0.63	0.11	15205.30	370.76	53	0.79	0.73	0.11	14861.30
Forward and reverse keyed items	342.36	53	0.72	0.65	0.11	15195.01	357.07	53	0.80	0.75	0.11	14847.61
Three facets	307.44	51	0.75	0.67	0.10	15172.47	513.27	51	0.69	0.60	0.13	15016.24
Three facets plus acquiescence	231.06	50	0.82	0.77	0.09	15102.27	316.08	50	0.82	0.76	0.10	14825.26
**Conscientiousness**												
Single domain	397.21	54	0.76	0.71	0.11	15635.85	586.80	54	0.71	0.64	0.14	15588.11
Single domain plus acquiescence	362.41	53	0.78	0.73	0.11	15607.24	323.29	53	0.85	0.81	0.10	15330.82
Forward and reverse keyed items	368.38	53	0.78	0.72	0.11	15613.21	323.11	53	0.85	0.82	0.10	15330.63
Three facets	217.37	51	0.88	0.85	**0.08**	15474.57	522.21	51	0.74	0.66	0.14	15542.17
Three facets plus acquiescence	161.57	50	**0.92**	**0.90**	**0.07**	15424.96	224.70	50	**0.90**	0.87	**0.08**	15250.87
**Negative emotionality**												
Single domain	372.43	54	0.87	0.84	0.11	15663.62	670.47	54	0.77	0.72	0.15	15631.82
Single domain plus acquiescence	349.66	53	0.88	0.85	0.11	15647.04	463.66	53	0.84	0.81	0.12	15431.23
Forward and reverse keyed items	344.69	53	0.88	0.85	0.11	15642.07	441.17	53	0.85	0.82	0.12	15408.74
Three facets	179.67	51	**0.95**	**0.93**	**0.07**	15489.42	473.92	51	0.84	0.79	0.13	15453.91
Three facets plus acquiescence	135.30	50	**0.96**	**0.95**	**0.06**	15451.24	200.19	50	**0.94**	**0.93**	**0.08**	15186.40
**Open-mindedness**												
Single domain	586.60	54	0.69	0.62	0.14	16166.99	742.84	54	0.63	0.54	0.16	16193.79
Single domain plus acquiescence	585.37	53	0.69	0.61	0.14	16171.95	675.15	53	0.66	0.58	0.15	16132.32
Forward and reverse keyed items	586.35	53	0.69	0.61	0.14	16172.93	718.78	53	0.64	0.55	0.16	16175.95
Three facets	133.45	51	**0.95**	**0.94**	**0.06**	15732.41	299.65	51	0.86	0.82	**0.10**	15769.25
Three facets plus acquiescence	112.42	50	**0.96**	**0.95**	**0.05**	15717.57	143.18	50	**0.95**	**0.93**	**0.06**	15618.99

CFI, comparative fit index; TLI, Tucker-Lewis index; RMSEA, root mean square error of approximation; BIC, Bayesian information criterion. CFI and TLI values ≥ 0.90 and RMSEA values ≤ 0.08 are in bold. The participants in the student and community sample included 487 and 500 Japanese adults, respectively.

### Reliability and validity

Table 5 presents Cronbach’s alpha and Omega in each survey and retest correlations between time 1 and 2 in the student sample for each domain and facet, as well as scale means and standard deviations. Cronbach’s alpha and Omega coefficients at the domain level were all above or near 0.80. At the facet level, coefficients tended to be low values; specifically, the coefficients of Trust in Agreeableness and Intellectual curiosity in Open-mindedness were under 0.60 in both samples. On the other hand, retest correlations between times for both the domains and facets ranged from 0.72 to 0.90. These results indicate that the BFI-2-J domain and facet scales have adequate internal consistency, as well as retest reliability.

**Table 5:** Descriptive statistics and reliability coefficients.

	Student				Community				*r* (test–retest)
	*M*	*SD*	*α*	*ω*	*M*	*SD*	*α*	*ω*	
Extraversion	2.98	0.72	0.89	0.90	2.72	0.68	0.87	0.88	0.89
Sociability	3.22	0.91	0.86	0.86	2.82	0.89	0.82	0.82	0.85
Assertiveness	2.63	0.78	0.74	0.76	2.52	0.78	0.71	0.72	0.84
Energy level	3.10	0.78	0.73	0.73	2.82	0.69	0.65	0.68	0.78
Agreeableness	3.31	0.48	0.74	0.74	3.26	0.50	0.79	0.79	0.85
Compassion	3.38	0.62	0.59	0.60	3.26	0.63	0.63	0.65	0.72
Respectfulness	3.55	0.57	0.51	0.47	3.52	0.64	0.62	0.62	0.72
Trust	2.98	0.66	0.58	0.58	3.00	0.56	0.53	0.54	0.78
Conscientiousness	2.77	0.58	0.82	0.82	3.18	0.58	0.84	0.83	0.85
Organization	2.85	0.79	0.73	0.74	3.26	0.70	0.67	0.68	0.74
Productiveness	2.73	0.70	0.65	0.66	3.20	0.71	0.67	0.65	0.76
Responsibility	2.72	0.64	0.63	0.64	3.07	0.64	0.62	0.62	0.74
Negative emotionality	3.13	0.73	0.89	0.89	3.06	0.69	0.89	0.89	0.90
Anxiety	3.33	0.83	0.77	0.78	3.24	0.81	0.76	0.77	0.80
Depression	3.14	0.81	0.71	0.73	3.04	0.78	0.73	0.75	0.83
Emotional volatility	2.92	0.87	0.81	0.82	2.89	0.79	0.80	0.80	0.85
Open-mindedness	3.28	0.60	0.81	0.81	3.08	0.57	0.80	0.81	0.85
Intellectual curiosity	3.50	0.66	0.59	0.59	3.21	0.58	0.43	0.43	0.80
Aesthetic sensitivity	3.29	0.96	0.82	0.83	2.99	0.89	0.82	0.83	0.88
Creative imagination	3.03	0.71	0.69	0.72	3.03	0.74	0.75	0.77	0.80

The term between the test and retest was three weeks. The participants in the student and community sample included 487 and 500 Japanese adults, respectively. The test-retest analysis samples were 171 university students in Tokyo.

Table 6 presents correlation coefficients between mean scores of each domain and facet on the BFI-2-J and mean scores of each domain on other Big Five measures (i.e., BFS-S, TIPI-J, FFPQ-50, BFPI, and NEO-FFI) to test convergent and discriminant validity. The five domains of the BFI-2-J were highly associated with the corresponding domains of other Big Five measures (*r* = 0.54–0.87, *M* = 0.72) apart from the association of Open-mindedness between the BFI-J and the BFPI (*r* = 0.29). The facets of the BFI-2-J were also highly associated with the convergent domains of each Big Five measure compared with the divergent domains. Interestingly, the BFI-2-J Open-Mindedness facets converged much more strongly with some Japanese Big Five measures (e.g., the FFPQ-50) than with others (e.g., the BFPI), suggesting that the definition of this domain varies across measures. The correlation coefficients between means of the domains and the facets in the BFI-2-J are shown in the Supplementary Table S6. Overall, these results indicate that the BFI-2-J shows good convergent and discriminant validity with other Japanese Big Five measures.

**Table 6:** Correlations of the BFI-2-J with the BFS-S, TIPI-J, FFPQ-50, BFPI, NEO-FFI, and RSES-J.

	BFS-S					TIPI-J					FFPQ-50					BFPI					NEO-FFI					RSES-J
	E	A	C	N	O	E	A	C	N	O	E	A	C	N	O	E	A	C	N	O	E	A	C	N	O
Extraversion	**0.85**	−0.06	−0.06	−0.43	0.46	**0.87**	−0.11	0.15	−0.19	0.37	**0.73**	0.32	0.09	−0.32	0.18	**0.85**	0.26	0.09	−0.09	0.38	**0.74**	0.14	0.24	−0.32	0.09	**0.60**
Sociability	**0.90**	−0.05	−0.10	−0.38	0.31	**0.88**	−0.14	0.05	−0.21	0.23	**0.66**	0.35	0.01	−0.28	0.09	**0.83**	0.24	−0.05	−0.23	0.23	**0.72**	0.24	0.13	−0.28	0.08	0.46
Assertiveness	**0.59**	−0.03	0.09	−0.41	0.39	**0.66**	−0.07	0.24	−0.22	0.35	**0.60**	0.18	0.18	−0.40	0.17	**0.75**	0.30	0.13	−0.04	**0.53**	0.44	−0.11	0.26	−0.35	0.11	**0.59**
Energy level	**0.71**	−0.07	−0.15	−0.33	**0.51**	**0.71**	−0.08	0.12	−0.07	0.42	**0.60**	0.29	0.06	−0.14	0.20	**0.62**	0.13	0.18	0.07	0.23	**0.71**	0.20	0.26	−0.19	0.03	**0.52**
Agreeableness	0.12	**0.61**	0.24	−0.31	0.14	0.00	**0.63**	0.18	−0.29	0.13	0.16	**0.74**	0.27	−0.31	−0.09	0.11	**0.61**	0.21	−0.25	0.01	0.40	**0.75**	0.33	−0.35	0.02	0.36
Compassion	0.19	0.34	0.27	−0.11	0.14	0.13	0.43	0.31	−0.16	0.09	0.18	**0.69**	0.25	−0.03	0.03	0.18	**0.62**	0.27	0.04	0.22	0.46	**0.68**	0.30	−0.26	0.01	0.33
Respectfulness	−0.08	**0.53**	0.25	−0.15	−0.03	−0.16	**0.58**	0.14	−0.20	−0.01	0.01	**0.55**	0.30	−0.20	−0.17	−0.10	0.29	0.26	−0.28	−0.12	0.15	**0.52**	0.33	−0.17	0.00	0.25
Trust	0.14	0.45	0.03	−0.39	0.18	0.03	0.38	−0.05	−0.26	0.20	0.18	**0.52**	0.11	**−0.50**	−0.08	0.13	0.46	−0.02	−0.34	−0.09	0.32	**0.62**	0.18	−0.42	0.03	0.31
Conscientiousness	0.00	0.26	**0.80**	0.03	0.05	−0.02	0.26	**0.66**	−0.05	−0.05	0.03	0.19	**0.84**	−0.20	−0.16	0.06	0.32	**0.73**	0.20	0.39	0.07	0.20	**0.76**	−0.39	0.12	**0.54**
Organization	−0.20	0.26	**0.70**	0.12	−0.05	−0.20	0.21	0.41	−0.06	−0.09	−0.14	0.13	**0.61**	0.01	−0.10	−0.08	0.21	**0.53**	0.28	0.22	−0.06	0.05	**0.55**	−0.30	0.12	0.34
Productiveness	0.21	0.14	0.40	−0.14	0.16	0.17	0.15	0.47	−0.09	0.00	0.13	0.20	**0.77**	−0.27	−0.11	0.21	0.37	**0.69**	0.05	0.40	0.19	0.30	**0.68**	−0.39	0.07	**0.54**
Responsibility	0.04	0.19	**0.68**	0.05	0.03	0.04	0.23	**0.64**	0.05	0.00	0.12	0.15	**0.73**	−0.28	−0.20	0.04	0.20	**0.60**	0.14	0.36	0.05	0.16	**0.63**	−0.25	0.11	**0.50**
Negative emotionality	−0.24	**−0.56**	0.03	**0.79**	−0.12	−0.17	−0.35	0.09	**0.77**	−0.08	−0.14	−0.21	−0.17	**0.82**	0.29	−0.19	−0.17	0.03	**0.78**	−0.01	−0.41	−0.38	−0.20	**0.70**	−0.14	**−0.63**
Anxiety	−0.25	−0.38	0.08	**0.79**	−0.15	−0.19	−0.24	0.11	**0.69**	−0.14	−0.09	−0.08	−0.08	**0.77**	0.26	−0.23	−0.07	0.09	**0.79**	0.00	−0.41	−0.37	−0.17	**0.66**	−0.07	**−0.56**
Depression	−0.31	−0.40	0.10	**0.75**	−0.13	−0.22	−0.27	0.14	**0.65**	−0.11	−0.21	−0.18	−0.05	**0.78**	0.23	−0.18	−0.12	0.12	**0.73**	0.00	**−0.51**	−0.35	−0.12	**0.67**	−0.11	**−0.65**
Emotional volatility	−0.10	**−0.70**	−0.11	**0.60**	−0.04	−0.04	−0.43	0.00	**0.71**	0.04	−0.07	−0.28	−0.30	**0.61**	0.27	−0.08	−0.28	−0.12	**0.55**	−0.04	−0.17	−0.29	−0.24	**0.55**	−0.19	−0.43
Open-mindedness	0.09	−0.02	−0.08	−0.03	**0.54**	0.11	0.03	−0.04	−0.06	**0.58**	−0.06	0.17	−0.07	0.26	**0.83**	0.05	0.07	0.05	0.25	0.29	0.12	−0.01	0.09	−0.18	**0.70**	0.43
Intellectual curiosity	0.05	−0.04	−0.03	0.08	0.43	0.05	−0.03	0.00	0.01	0.40	0.01	0.15	0.04	0.30	**0.66**	0.11	0.18	0.19	0.28	0.39	−0.02	−0.17	0.04	−0.09	**0.66**	0.26
Aesthetic sensitivity	0.01	−0.05	−0.01	0.07	0.28	0.00	0.00	−0.01	0.02	0.27	−0.20	0.06	−0.13	0.25	**0.65**	−0.17	0.01	−0.04	0.28	−0.02	0.01	−0.02	0.02	−0.08	**0.66**	0.19
Creative imagination	0.17	0.06	−0.15	−0.22	**0.52**	0.20	0.09	−0.09	−0.19	**0.65**	0.12	0.20	−0.05	0.02	**0.58**	0.26	−0.03	0.00	−0.03	0.39	0.33	0.14	0.16	−0.28	0.30	**0.57**

Absolute correlation coefficients ≥0.50 are in bold. The respondents to the BFS-S, TIPI-J, FFPQ-50, BFPI, and NEO-FFI were 150, 152, 87, and 119 university students, respectively. There were 500 respondents to the RSES-J.

Table 6 also presents the correlation coefficients between the BFI-2-J and RSES-J. As expected, high Extraversion and low Negative Emotionality correlated strongly with self-esteem at both the domain and facet levels (approximately |*r*| > 0.50). Moreover, self-esteem’s correlation coefficient with Conscientiousness was higher than with Open-mindedness or Agreeableness. Open-mindedness, and Agreeableness moderately correlated with self-esteem. These results were very consistent with expectations from previous research on personality traits and self-esteem, and further support the validity of the BFI-2-J.

### Measurement invariance

Our final set of analyses examined MI across age and sex groups in the domain-level and facet-level models in the community sample. The domain-level model adopted exploratory structural equation modeling (ESEM) (Asparouhov and Muthén, 2009) using the mean scores of each of the 15 facets because there were several cross-loadings at the domain level. The domain-level model with ESEM consisted of all facets loaded on all five domains with EFA (varimax rotation, maximum likelihood method); all facets had their strongest loading on their intended primary factor (Supplementary Table S7). The facet-level models adopted the three facets plus acquiescence model, described above, within each Big Five domain.

Tables 7, 8, present the results of the MI analyses across age and sex groups. We adopted a decrease in CFI of no more than 0.01 and an increase in RMSEA of no more than 0.015 as the standards for establishing MI (Chen, 2007). The CFI values indicate that the test of strong invariance resulted in fit similar to metric invariance models with the exception of MI across age groups for the Open-Mindedness facet-level model and MI across sex groups for the domain-level model, as well as the Extraversion, and Agreeableness facet-level models. The RMSEA values indicate no more than modest decreases in fit at stricter levels of MI for both the domain and facet-level models. Overall, these results suggest that scores from the BFI-2-J are strictly comparable across sex and age groups at the level of the Big Five domains, and strongly comparable at the facet level.

**Table 7:** Fit statistics for measurement invariance analyses at each age group.

	*χ^2^*	*df*	CFI	TLI	RMSEA	BIC	ΔCFI	ΔRMSEA
**Domain using facet scores**								
Configural/Metric	307.97	180	**0.97**	**0.96**	**0.05**	13058.45		
Strong	329.05	190	**0.96**	**0.96**	**0.05**	13017.39	−0.003	0.001
Strict	395.02	205	**0.95**	**0.95**	**0.06**	12990.14	−0.014	0.007
**Extraversion**								
Configural	436.93	100	0.87	0.83	0.12	15733.85		
Metric	461.64	109	0.86	0.84	0.11	15702.63	−0.006	−0.002
Strong	474.48	117	0.86	0.85	0.11	15665.75	−0.002	−0.003
Strict	559.58	129	0.83	0.83	0.12	15676.28	−0.028	0.005
**Agreeableness**								
Configural	389.53	100	0.81	0.75	0.11	14889.51		
Metric	409.97	109	0.81	0.76	0.11	14854.02	−0.007	−0.003
Strong	426.79	117	0.80	0.77	0.10	14821.12	−0.006	−0.002
Strict	496.68	129	0.76	0.76	0.11	14816.43	−0.037	0.004
**Conscientiousness**								
Configural	276.06	100	**0.91**	0.88	**0.08**	15293.24		
Metric	299.46	109	**0.90**	0.88	**0.08**	15260.71	−0.008	0.000
Strong	302.74	117	**0.90**	0.89	**0.08**	15214.28	0.003	−0.004
Strict	386.19	129	0.86	0.86	**0.09**	15223.16	−0.038	0.010
**Negative emotionality**								
Configural	278.46	100	**0.93**	**0.91**	**0.08**	15298.64		
Metric	288.51	109	**0.93**	**0.92**	**0.08**	15252.76	0.000	−0.003
Strong	301.11	117	**0.93**	**0.92**	**0.08**	15215.64	−0.002	−0.002
Strict	346.56	129	**0.92**	**0.92**	**0.08**	15186.51	−0.013	0.003
**Open-mindedness**								
Configural	204.81	100	**0.94**	**0.93**	**0.06**	15723.12		
Metric	208.39	109	**0.95**	**0.94**	**0.06**	15670.77	0.003	−0.004
Strong	246.87	117	**0.93**	**0.92**	**0.07**	15659.53	−0.016	0.006
Strict	276.75	129	**0.92**	**0.92**	**0.07**	15614.84	−0.010	0.001

CFI, comparative fit index; TLI, Tucker-Lewis index; RMSEA, root mean square error of approximation; BIC, Bayesian information criterion. CFI and TLI values ≥ 0.90 and RMSEA values ≤0.08, are in bold. The sample was divided into two age groups: 44 years or younger (*n* = 239) and 45 years or older (*n* = 261). The fit statistics on the domain model have the same values between the configural and metric models because the ESEM was analyzed with the loadings on the EFA. The analyses of MI in each domain are used in the model of the three facets plus acquiescence.

**Table 8:** Fit statistics for measurement invariance analyses at each sex group.

	*χ^2^*	*df*	CFI	TLI	RMSEA	BIC	ΔCFI	ΔRMSEA
**Domain using facet scores**								
Configural/Metric	365.31	180	**0.95**	**0.94**	**0.06**	13070.92		
Strong	440.84	190	**0.93**	**0.93**	**0.07**	13084.30	−0.017	0.008
Strict	465.62	205	**0.93**	**0.93**	**0.07**	13015.87	−0.003	−0.001
**Extraversion**								
Configural	393.72	100	0.88	0.85	0.11	15771.52		
Metric	411.18	109	0.88	0.85	0.11	15733.05	−0.003	−0.003
Strong	473.83	117	0.86	0.84	0.11	15745.98	−0.022	0.005
Strict	490.01	129	0.86	0.85	0.11	15687.59	−0.002	−0.005
**Agreeableness**								
Configural	372.15	100	0.83	0.77	0.10	14931.60		
Metric	406.29	109	0.81	0.77	0.10	14909.81	−0.016	0.000
Strong	434.78	117	0.80	0.77	0.10	14888.58	−0.013	0.000
Strict	475.91	129	0.78	0.77	0.10	14855.14	−0.019	−0.001
**Conscientiousness**								
Configural	283.09	100	**0.90**	0.87	**0.09**	15412.96		
Metric	302.30	109	**0.90**	0.87	**0.08**	15376.25	−0.005	−0.001
Strong	318.63	117	0.89	0.88	**0.08**	15342.86	−0.004	−0.001
Strict	336.34	129	0.89	0.89	**0.08**	15286.00	−0.003	−0.003
**Negative emotionality**								
Configural	268.69	100	**0.94**	**0.92**	**0.08**	15362.30		
Metric	272.24	109	**0.94**	**0.93**	**0.08**	15309.92	0.002	−0.005
Strong	283.39	117	**0.94**	**0.93**	**0.08**	15271.36	−0.001	−0.002
Strict	296.39	129	**0.94**	**0.94**	**0.07**	15209.78	0.000	−0.003
**Open-mindedness**								
Configural	214.36	100	**0.94**	**0.92**	**0.07**	15760.12		
Metric	219.25	109	**0.94**	**0.93**	**0.06**	15709.09	0.002	−0.004
Strong	224.32	117	**0.94**	**0.94**	**0.06**	15664.44	0.002	−0.003
Strict	244.90	129	**0.94**	**0.94**	**0.06**	15610.44	−0.005	−0.001

CFI, comparative fit index; TLI, Tucker-Lewis index; RMSEA, root mean square error of approximation; BIC, Bayesian information criterion. CFI and TLI values ≥ 0.90 and RMSEA values ≤ 0.08, are in bold. The fit statistics on the domain model have the same values between the configural and metric models because the ESEM was analyzed with the loadings on the EFA. The analyses of MI in each domain are used in the model of the three facets plus acquiescence.

After confirming at least strong MI of the BFI-2-J, we examined the mean-level differences between age and sex groups. Table 9 presents the mean-level differences for age (<45 versus ≥45 years) and sex. These results indicate that older people tended to score moderately lower in Negative Emotionality, as well as higher in Conscientiousness, Agreeableness, and Open-Mindedness, than younger people; these age differences extended to most but not all facets within these domains. Table 9 also shows that sex differences were generally small, although at the facet level, females tended to score moderately higher on Aesthetic sensitivity, and lower on Assertiveness, than males.

**Table 9:** Descriptive statistics at each age and sex group.

	44 years or younger		45 years or older		Cohen’s *d*	Female		Male		Cohen’s *d*
	*M*	*SD*	*M*	*SD*		*M*	*SD*	*M*	*SD*	
Extraversion	2.66	0.70	2.76	0.65	−0.15	2.68	0.65	2.75	0.71	−0.10
Sociability	2.75	0.95	2.88	0.83	−0.15	2.89	0.85	2.74	0.92	0.17
Assertiveness	2.45	0.76	2.58	0.78	−0.17	2.36	0.72	2.68	0.80	−0.42
Energy level	2.80	0.72	2.83	0.67	−0.05	2.80	0.68	2.83	0.71	−0.04
Agreeableness	3.19	0.51	3.33	0.49	−0.28	3.29	0.51	3.23	0.49	0.13
Compassion	3.21	0.66	3.30	0.60	−0.14	3.31	0.61	3.21	0.64	0.17
Respectfulness	3.42	0.69	3.61	0.57	−0.29	3.55	0.61	3.48	0.66	0.11
Trust	2.92	0.57	3.07	0.55	−0.26	3.01	0.59	2.99	0.54	0.03
Conscientiousness	3.09	0.59	3.26	0.57	−0.30	3.17	0.57	3.18	0.59	−0.02
Organization	3.21	0.75	3.31	0.65	−0.13	3.25	0.71	3.27	0.69	−0.04
Productiveness	3.08	0.70	3.31	0.69	−0.33	3.17	0.68	3.23	0.72	−0.07
Responsibility	2.97	0.67	3.17	0.61	−0.32	3.10	0.63	3.05	0.65	0.08
Negative emotionality	3.19	0.70	2.94	0.66	0.36	3.11	0.70	3.00	0.68	0.15
Anxiety	3.34	0.82	3.15	0.78	0.24	3.30	0.79	3.18	0.82	0.15
Depression	3.18	0.79	2.92	0.75	0.33	3.07	0.79	3.02	0.77	0.08
Emotional volatility	3.04	0.82	2.75	0.74	0.38	2.96	0.79	2.82	0.79	0.17
Open-mindedness	2.99	0.58	3.15	0.56	−0.29	3.08	0.56	3.07	0.58	0.01
Intellectual curiosity	3.21	0.62	3.22	0.54	−0.02	3.15	0.53	3.27	0.62	−0.21
Aesthetic sensitivity	2.88	0.93	3.09	0.84	−0.23	3.12	0.90	2.86	0.86	0.30
Creative imagination	2.89	0.73	3.16	0.73	−0.37	2.97	0.71	3.09	0.77	−0.17

## Discussion

This study aimed to adapt the BFI-2-J which was translated from the original, English-language version of the BFI-2. We investigated the BFI-2-J for factor structure at the domain and facet levels, as well as reliability, validity, and measurement invariance across age and sex groups. The overall results of these analyses using two datasets provided evidence that the BFI-2-J provides reliable and valid assessment of Big Five domains and facets in the Japanese language and cultural context. Given the increasingly widespread use of the BFI-2, we conclude that the BFI-2-J is more suitable for international and cross-cultural research than original Japanese measures.

We found that the domain-level structure of the BFI-2-J was nearly the same as in the source version of the BFI-2. We also examined facet-level structures within each Big Five domain. These results supported that the model of the three facets plus acquiescence indicated an acceptable fit to both datasets within each Big Five domain. Strictly, the fit statistics in Extraversion and Agreeableness did not fully reach the criterion level. In order to improve the fit statistics of the facet-level structure within Extraversion, it was necessary to add one error covariance between items 16 and 46, which both emphasize talkativeness, to the three facets plus acquiescence model. In contrast, the fit statistics of the facet-level structure within Agreeableness did not improve by including error co-variances *ad hoc*. We speculate that Japanese phrases and words related to Agreeableness may be ambiguous compared to other languages. For example, being “polite” may mean obeying social rule in Japan, rather than respecting an individual social partner. Therefore, it is necessary to examine the existence of unique Japanese structures related to this Big Five domain. Few studies have investigated the hierarchical structure of Japanese personality traits, apart from studies on the development of the measures. In parallel with the improvement of items of the BFI-2-J, we should proceed with further lexical research to better understand the facet-level structure of Agreeableness in Japan.

Regarding reliability, the BFI-2-J was sufficiently reliable to assess individual differences among persons in terms of the Big Five personality traits. The test–retest correlations were over 0.72, similar to those reported by Soto and John (2017), though it is necessary to note that the interval between times 1 and 2 was a little short. Some internal consistencies were moderately low at the facet level, although those at the domain level were acceptable. However, there were only four items within a facet, and these values are similar to other BFI-2 translations (Shchebetenko et al., 2020; Zhang et al., 2021); therefore, it is necessary to evaluate the utility of the BFI-2-J from the perspective of associations with other criteria given below.

In terms of convergent and discriminant validity, the BFI-2-J produced the expected results. The correlations between the domains of the BFI-2-J and the convergent domains of the other Big Five measures were higher than that of the divergent domains. Moreover, correlations with the facets of BFI-2-J usually indicated a similar pattern to the domains. However, there were a few lower correlation coefficients between convergent pairs for Open-Mindedness. As we can see from the different labels that are sometimes used for this domain (e.g., Openness, Intellect), the concept and definition of Open-Mindedness is less agreed upon than for other domains (DeYoung, 2014). Reflecting this diversity of conceptualizations, Open-Mindedness in the BFI-2 covers a wide range of concepts by designing the three facets without deciding a central facet within this domain (Soto and John, 2017). In contrast, other Japanese Big Five measures appear to adopt different conceptualizations; for example, several exclude content related to the BFI-2 facet of Aesthetic sensitivity.

Further supporting the construct validity of the BFI-2-J, correlations with self-esteem were consistent with hypotheses derived from previous research regarding personality traits and self-esteem. Most notably, self-esteem correlated more strongly with Extraversion and Negative Emotion than with the other traits.

Regarding measurement invariance, we confirmed that a domain-level model showed strict MI across age groups, as well as metric MI across sex groups. Moreover, the facet-level models within each Big Five domain all showed at least strong MI across age groups, and most also showed at least strong MI across sex groups. We also reported mean-level differences between age and sex groups. The results of age difference converged with findings from previous personality development research (Soto et al., 2011). Interestingly, the effect sizes obtained here were large compared with a previous study of the BFI-2 in the Russian context (Shchebetenko et al., 2020), possibly reflecting cultural variation or differences between the age cutoffs used in these two studies (25 versus 45 years). Additionally, some results differed depending on facets within a domain; for example, males had a higher mean Assertiveness score than females (*d* = 0.42), while females had a higher Sociability score (*d* = 0.17). Age, sex, and other demographic differences in personality traits in Japan can be further investigated in future research using the BFI-2-J.

Finally, the present findings provide further evidence regarding the importance of accounting for individual differences in acquiescent response style in questionnaire measures (Danner et al., 2015; Soto and John, 2019). In terms of observed scale scores, the fact that each BFI-2 domain and facet scale includes an equal number of forward-keyed and reverse-keyed items means that their observed scale scores automatically control for acquiescent responding: each respondent’s bias toward agreeing (or disagreeing) with the forward-keyed items will be offset by their bias toward agreeing (or disagreeing) with the false-keyed items (Soto and John, 2017). At the item level, however, we found that adequately modeling the BFI-2-J items required the inclusion of a latent variable representing acquiescence. Including this acquiescence method factor substantially improved model fit for the items within each Big Five domain. We therefore encourage other researchers who wish to model item-level responses on measures that include both forward-keyed and reverse-keyed items to similarly account for acquiescent response style (Danner et al., 2021).

### Limitations and future directions

The present findings indicate that the Japanese version of the BFI-2 can be used at both the domain and facet levels to some extent; however, this study had some limitations that highlight directions for future research. First, the BFI-2-J can be used to examine associations with additional life outcomes (Soto, 2019; Denissen et al., 2020; Cemalcilar et al., 2021). This study investigated whether the Big Five personality traits are associated with only self-esteem as a criterion other than the Big Five measures. We therefore need to examine associations with other dimensions of well-being or a broader range of external criteria for predictive validity. Such studies would contribute not only to the utility of the BFI-2-J but also to the literature on non-WEIRD findings. For example, Cemalcilar et al. (2021) found that Agreeableness, Extraversion, and Open-Mindedness negatively predicted religiosity in Turkey, where most of the population is Muslim, while there were positive associations of religious belief with Agreeableness and Conscientiousness in the United States (Soto, 2019). It may be similarly possible to find the specific characteristics of the Japanese population; for example, smokers had higher Extraversion levels than never smokers in Japan (Abe et al., 2019).

Second, validity at the facet level should be further examined. Although this study recovered facet structures within each Big Five domain, correlation coefficients of facet scores were only examined using the domain scores of other Big Five scales. Previous studies supported convergence and discrimination at the facet level by testing correlations between the facets of the BFI-2 and NEO-PI-R (Costa and McCrae, 1992), which includes six facets per Big Five domain (Soto and John, 2017; Rammstedt et al., 2020). Moreover, previous research has indicated that facet-level traits can predict several outcomes more strongly than domains because a facet covers a narrower and more specific trait concept (Paunonen and Ashton, 2001). As per Soto and John (2017), we expect that the proportion of criterion variance explained by 15 facets is higher than that explained by five domains. However, additional research is needed to test this hypothesis.

### Conclusion

In sum, the present research indicates that the BFI-2-J is a reliable, valid, efficient, and freely available measure of the Big Five domains and facets in the Japanese language and cultural context. We therefore expect that this measure will be widely used in Japanese personality research. Moreover, there is a growing body of literature on adapting the BFI-2 in several languages. This work enables a comparison of factor structure, associations with life outcomes across cultural areas, and geographical differences in personality in future research.

## Data availability statement

The datasets generated for this study are available on request to the corresponding author. The corresponding author can be contacted regarding the dataset, the survey, and the analysis.

## Ethics statement

The studies involving human participants were reviewed and approved by the Ethics Review of Committee on Research with Human Subjects in Waseda University. Written informed consent for participation was not required for this study in accordance with the national legislation and the institutional requirements.

## Author contributions

TSh, AO, YH, YU, and TM conceived the study design; SY, TSh, AO, YH, YU, TM, and IM translated the original version of the scale; SY, TSh, AO, YH, YU, TM, IM TSa, and SK collected the data; SY and TSh performed the statistical analysis; SY, TSh, and AO wrote the first draft of the manuscript; CJS critically revised the manuscript; All authors contributed to the final manuscript.

## Funding

This work was supported by JSPS (Japan Society for the Promotion of Science) KAKENHI grant numbers 17 K04376 and 20 K03345.

## Conflict of interest

The authors declare that the research was conducted in the absence of any commercial or financial relationships that could be construed as a potential conflict of interest.

## Publisher’s note

All claims expressed in this article are solely those of the authors and do not necessarily represent those of their affiliated organizations, or those of the publisher, the editors and the reviewers. Any product that may be evaluated in this article, or claim that may be made by its manufacturer, is not guaranteed or endorsed by the publisher.

## Footnotes

^1^ https: //www.agekke.co.jp/

^2^ https://www.stat.go.jp/data/kokusei/2020/index.html

## References

[ref1] Abe S. Oshio A. Kawamoto T. Ito H. Hirashima T. Tsubota Y.. (2019). Smokers are extraverted in Japan: smoking habit and the big five personality traits. SAGE Open 9:215824401985995. doi: 10.1177/2158244019859956

[ref2] Aoki T. (1971). A psycho-lexical study of personality trait words. Jpn. J. Psychol. 42, 1–13. doi: 10.4992/jjpsy.42.1

[ref3] Ashton M. C. Lee K. (2007). Empirical, theoretical, and practical advantages of the HEXACO model of personality structure. Personal. Soc. Psychol. Rev. 11, 150–166. doi: 10.1177/1088868306294907, PMID: 18453460

[ref4] Asparouhov T. Muthén B. (2009). Exploratory structural equation modeling. Struct. Equ. Model. Multidiscip. J. 16, 397–438. doi: 10.1080/10705510903008204

[ref5] Cemalcilar Z. Baruh L. Kezer M. Soto C. J. Sumer N. John O. P. (2021). Testing the BFI-2 in a non-WEIRD community sample. Pers. Individ. Dif. 182:111087. doi: 10.1016/j.paid.2021.111087

[ref6] Chen F. F. (2007). Sensitivity of goodness of fit indexes to lack of measurement invariance. Struct. Equ. Model. Multidiscip. J. 14, 464–504. doi: 10.1080/10705510701301834

[ref7] Costa P. T. McCrae R. R. (1992). NEO-PI-R Professional Manual: Revised NEO Personality Inventory (NEO-PI-R) and NEO Five-Factor Inventory (NEO-FFI). Odessa, FL: Psychological Assessment Resources.

[ref8] Danner D. Aichholzer J. Rammstedt B. (2015). Acquiescence in personality questionnaires: relevance, domain specificity, and stability. J. Res. Pers. 57, 119–130. doi: 10.1016/j.jrp.2015.05.004

[ref9] Danner D. Lechner C. M. Soto C. J. John O. P. (2021). Modelling the incremental value of personality facets: the domains-incremental facets-acquiescence bifactor showmodel. Eur. J. Personal. 35, 67–84. doi: 10.1002/per.2268

[ref10] Danner D. Rammstedt B. Bluemke M. Treiber L. Berres S. Soto C.. (2016). *Die Deutsche Version des Big Five Inventory 2 (BFI-2)*. *Zusammenstellung sozialwissenschaftlicher Items und Skalen [German version of the Big Five Inventory 2 (BFI-2)]* (Mannheim, Germany: GESIS). doi:10.6102/zis247.

[ref11] Denissen J. J. A. Geenen R. Soto C. J. John O. P. Van Aken M. A. G. (2020). The big five Inventory-2: replication of psychometric properties in a Dutch adaptation and first evidence for the discriminant predictive validity of the facet scales. J. Pers. Assess. 102, 309–324. doi: 10.1080/00223891.2018.1539004, PMID: 30638406

[ref12] De Raad B. Barelds D. P. Levert E. Ostendorf F. Mlačić B. Blas L. D.. (2010). Only three factors of personality description are fully replicable across languages: a comparison of 14 trait taxonomies. J. Pers. Soc. Psychol. 98, 160–173. doi: 10.1037/a0017184, PMID: 20053040

[ref13] DeYoung C. G. (2014). “Openness/intellect: a dimension of personality reflecting cognitive exploration,” in APA Handbook of Personality and Social Psychology: Personality Processes and Individual Differences. eds. Cooper M. L. Larsen R. J., vol. 4 (Washington, DC: American Psychological Association), 369–399.

[ref14] DeYoung C. G. Quilty L. C. Peterson J. B. (2007). Between facets and domains: 10 aspects of the big five. J. Pers. Soc. Psychol. 93, 880–896. doi: 10.1037/0022-3514.93.5.880, PMID: 17983306

[ref15] Føllesdal H. Soto C. J. (2022). The Norwegian adaptation of the big five Inventory-2. Front. Psychol. 13:858920. doi: 10.3389/fpsyg.2022.858920, PMID: 35664220 PMC9158541

[ref16] Fossati A. Borroni S. Marchione D. Maffei C. (2011). The big five inventory (BFI). Eur. J. Psychol. Assess. 27, 50–58. doi: 10.1027/1015-5759/a000043

[ref17] Fujishima Y. Yamada N. Tsuji H. (2005). Construction of short form of five factor personality questionnaire. Jpn. J. Pers. 13, 231–241. doi: 10.2132/personality.13.231

[ref18] Gebauer J. E. Sedikides C. Wagner J. Bleidorn W. Rentfrow P. J. Potter J.. (2015). Cultural norm fulfillment, interpersonal belonging, or getting ahead? A large-scale cross-cultural test of three perspectives on the function of self-esteem. J. Pers. Soc. Psychol. 109, 526–548. doi: 10.1037/pspp0000052, PMID: 26167799

[ref19] Goldberg L. R. (1981). “Language and individual differences: the search for universals in personality lexicons,” in Review of Personality and Social Psychology. ed. Wheeler L., vol. 2 (Beverly Hills: Sage), 141–165.

[ref20] Goldberg L. R. (1993). The structure of phenotypic personality traits. Am. Psychol. 48, 26–34. doi: 10.1037/0003-066X.48.1.26 8427480

[ref21] Gosling S. D. Rentfrow P. J. Swann W. B. Jr. (2003). A very brief measure of the big-five personality domains. J. Res. Pers. 37, 504–528. doi: 10.1016/S0092-6566(03)00046-1

[ref22] Halama P. Kohút M. Soto C. J. John O. P. (2020). Slovak adaptation of the big five inventory (BFI-2): psychometric properties and initial validation. Stud. Psychol. 62, 74–87. doi: 10.31577/sp.2020.01.792

[ref23] Hashimoto Y. (2018). A psycho-lexical study of Japanese interpersonal trait words. Bull. Grad. Div. lett. Arts Sci. Waseda Univ. 63, 39–54. https://www.waseda.jp/flas/glas/assets/uploads/2018/03/Vol63_HASHIMOTO-Yasuhiro_0039-0054.pdf

[ref24] Henrich J. Heine S. J. Norenzayan A. (2010). The weirdest people in the world? Behav. Brain Sci. 33, 61–83, discussion. doi: 10.1017/S0140525X0999152X, PMID: 20550733

[ref25] Hofstee W. K. de Raad B. Goldberg L. R. (1992). Integration of the big five and circumplex approaches to trait structure. J. Pers. Soc. Psychol. 63, 146–163. doi: 10.1037/0022-3514.63.1.146, PMID: 1494982

[ref26] John O. P. Naumann L. P. Soto C. J. (2008). “Paradigm shift to the integrative big five trait taxonomy. History, measurement, and conceptual issues,” in Handbook of Personality Theory and Research. eds. John O. P. Robins R. W. Pervin L. A.. 3rd ed (New York: Guilford Press), 114–159.

[ref27] John O. P. Srivastava S. (1999). “The big five trait taxonomy: history, measurement, and theoretical perspectives,” in Handbook of Personality: Theory and Research. eds. Pervin L. A. John O. P. (New York: Guilford Press), 102–138.

[ref28] Kashiwagi S. Tsuji H. Fujishima Y. Yamada N. (2005). Re-evaluation of Tsuji’s psycholexical study in terms of the big five personality factors. Jpn. J. Psychol. 76, 368–374. doi: 10.4992/jjpsy.76.368, PMID: 16318295

[ref29] Lee D. I. Gardiner G. Baranski E. Members of the International Situations Project Funder D. C. (2020). Situational experience around the world: a replication and extension in 62 countries. J. Pers. 88, 1091–1110. doi: 10.1111/jopy.12558, PMID: 32428272

[ref30] Marsh H. W. Hau K. T. Wen Z. (2004). In search of golden rules: comment on hypothesis-testing approaches to setting cutoff values for fit indexes and dangers in overgeneralizing Hu and Bentler's (1999) findings. Struct. Equ. Model. Multidiscip. J. 11, 320–341. doi: 10.1207/s15328007sem1103_2

[ref31] McCrae R. R. Terracciano A. 78 Members of the Personality Profiles of Cultures Project (2005a). Universal features of personality traits from the observer’s perspective: data from 50 cultures. J. Pers. Soc. Psychol. 88, 547–561. doi: 10.1037/0022-3514.88.3.547, PMID: 15740445

[ref32] McCrae R. R. Terracciano A. 79 Members of the Personality Profiles of Cultures Project (2005b). Personality profiles of cultures: aggregate personality traits. J. Pers. Soc. Psychol. 89, 407–425. doi: 10.1037/0022-3514.89.3.407, PMID: 16248722

[ref33] Murakami Y. (2002). A collection of basic personality trait words. Jpn. J. Pers. 11, 35–49. doi: 10.2132/jjpjspp.11.1_35

[ref34] Murakami Y. (2003). Big five and psychometric conditions for their extraction in Japanese. Jpn. J. Pers. 11, 70–85. doi: 10.2132/jjpjspp.11.2_70

[ref35] Murakami Y. Murakami T. (1999). Manual of big five. Tokyo: Gakugeitosho.

[ref36] Namikawa T. Tani I. Wakita T. Kumagai R. Nakane A. Noguchi H. (2012). Development of a short form of the Japanese big-five scale, and a test of its reliability and validity. Japan. J. Psychol. 83, 91–99. doi: 10.4992/jjpsy.83.91, PMID: 22834085

[ref37] Norman W. T. (1963). Toward an adequate taxonomy of personality attributes: replicated factor structure in peer nomination personality ratings. J. Abnorm. Soc. Psychol. 66, 574–583. doi: 10.1037/h0040291 13938947

[ref38] Oshio A. Abe S. Cutrone P. (2012). Development, reliability, and validity of the Japanese version of ten item personality inventory (TIPI-J). Jpn. J. Pers. 21, 40–52. doi: 10.2132/personality.21.40

[ref39] Oshio A. Abe S. Cutrone P. Gosling S. D. (2013). Big five content representation of the Japanese version of the ten-item personality inventory. Psychology 04, 924–929. doi: 10.4236/psych.2013.412133

[ref40] Oshio A. Abe S. Cutrone P. Gosling S. D. (2014). Further validity of the Japanese version of the ten item personality inventory (TIPI-J): cross-language evidence for content validity. J. Individ. Differ. 35, 236–244. doi: 10.1027/1614-0001/a000145

[ref41] Ozer D. J. Benet-Martínez V. (2006). Personality and the prediction of consequential outcomes. Annu. Rev. Psychol. 57, 401–421. doi: 10.1146/annurev.psych.57.102904.190127, PMID: 16318601

[ref42] Paunonen S. V. Ashton M. C. (2001). Big five factors and facets and the prediction of behavior. J. Pers. Soc. Psychol. 81, 524–539. doi: 10.1037/0022-3514.81.3.524, PMID: 11554651

[ref43] R Core Team. (2019). R: A Language And Environment For Statistical Computing. Vienna, Austria: R Foundation for Statistical Computing. Available at: https://www.r-project.org/

[ref44] Rammstedt B. Danner D. Soto C. J. John O. P. (2020). Validation of the short and extra-short forms of the big five Inventory-2 (BFI-2) and their German adaptations. Eur. J. Psychol. Assess. 36, 149–161. doi: 10.1027/1015-5759/a000481

[ref45] Rentfrow P. J. Gosling S. D. Potter J. (2008). A theory of the emergence, persistence, and expression of geographic variation in psychological characteristics. Perspect. Psychol. Sci. 3, 339–369. doi: 10.1111/j.1745-6924.2008.00084.x, PMID: 26158954

[ref46] Roberts B. W. Kuncel N. R. Shiner R. Caspi A. Goldberg L. R. (2007). The power of personality: the comparative validity of personality traits, socioeconomic status, and cognitive ability for predicting important life outcomes. Perspect. Psychol. Sci. 2, 313–345. doi: 10.1111/j.1745-6916.2007.00047.x, PMID: 26151971 PMC4499872

[ref47] Robins R. W. Tracy J. L. Trzesniewski K. Potter J. Gosling S. D. (2001). Personality correlates of self-esteem. J. Res. Pers. 35, 463–482. doi: 10.1006/jrpe.2001.2324

[ref48] Rosenberg M. (1965). Society and the adolescent self-image. Princeton, NJ: Prinston University Press. doi: 10.1515/9781400876136.

[ref49] Sakurai S. (2000). Investigation of the Japanese version of Rosenberg’s self-esteem scale. Bull. Tsukuba Dev. Clin. Psychol. 12, 65–71.

[ref50] Saucier G. Ostendorf F. (1999). Hierarchical subcomponents of the big five personality factors: a cross-language replication. J. Pers. Soc. Psychol. 76, 613–627. doi: 10.1037/0022-3514.76.4.613, PMID: 10234848

[ref51] Schmitt D. P. Allik J. McCrae R. R. Benet-Martínez V. (2007). The geographic distribution of big five personality traits: patterns and profiles of human self-description across 56 nations. J. Cross-Cult. Psychol. 38, 173–212. doi: 10.1177/0022022106297299

[ref52] Shchebetenko S. Kalugin A. Y. Mishkevich A. M. Soto C. J. John O. P. (2020). Measurement invariance and sex and age differences of the big five inventory–2: evidence from the Russian version. Assessment 27, 472–486. doi: 10.1177/1073191119860901, PMID: 31286794

[ref53] Shimonaka Y. Nakazato K. Gondo Y. Takayama M. (1999). Revised NEO-Personality Inventory (NEO-PI-R) and NEO Five Factor Inventory (NEO-FFI) Manual for the Japanese Version. Tokyo: Tokyo Shinri.

[ref54] Sleep C. E. Lynam D. R. Miller J. D. (2021). A comparison of the validity of very brief measures of the big five/five-factor model of personality. Assessment 28, 739–758. doi: 10.1177/1073191120939160, PMID: 32762351

[ref55] Soto C. J. (2019). How replicable are links between personality traits and consequential life outcomes? The life outcomes of personality replication project. Psychol. Sci. 30, 711–727. doi: 10.1177/0956797619831612, PMID: 30950321

[ref56] Soto C. J. (2021). Do links between personality and life outcomes generalize? Testing the robustness of trait-outcome associations across gender, age, ethnicity, and analytic approaches. Soc. Psychol. Personal. Sci. 12, 118–130. doi: 10.1177/1948550619900572

[ref57] Soto C. J. John O. P. (2009). Ten facet scales for the big five inventory: convergence with NEO PI-R facets, self-peer agreement, and discriminant validity. J. Res. Pers. 43, 84–90. doi: 10.1016/j.jrp.2008.10.002

[ref58] Soto C. J. John O. P. (2017). The next big five inventory (BFI-2): developing and assessing a hierarchical model with 15 facets to enhance bandwidth, fidelity, and predictive power. J. Pers. Soc. Psychol. 113, 117–143. doi: 10.1037/pspp0000096, PMID: 27055049

[ref59] Soto C. J. John O. P. (2019). Optimizing the length, width, and balance of a personality scale: how do internal characteristics affect external validity? Psychol. Assess. 31, 444–459. doi: 10.1037/pas0000586, PMID: 29792501

[ref60] Soto C. J. John O. P. Gosling S. D. Potter J. (2011). Age differences in personality traits from 10 to 65: big five domains and facets in a large cross-sectional sample. J. Pers. Soc. Psychol. 100, 330–348. doi: 10.1037/a0021717, PMID: 21171787

[ref61] Study Group of the FFPQ. (1998). FFPQ manual (in Japanese). Kyoto: Kitaohji shobo.

[ref62] Tsuji H. (2001). *Analysis for the Dimensions of Personality Based on Japanese Psycholexical Approach*. *Final Research Report in Grant-In-Aid for Scientific Research Program (KAKENHI).* Tokyo: The Ministry of Education, Culture, Sports, Science and Technology (MEXT).

[ref63] Tupes E. C. Christal R. E. (1992). Recurrent personality factors based on trait ratings. J. Pers. 60, 225–251. doi: 10.1111/j.1467-6494.1992.tb00973.x, PMID: 1635043

[ref64] Vazsonyi A. T. Ksinan A. Mikuška J. Jiskrova G. (2015). The big five and adolescent adjustment: an empirical test across six cultures. Pers. Individ. Dif. 83, 234–244. doi: 10.1016/j.paid.2015.03.049

[ref65] Vedel A. Wellnitz K. B. Ludeke S. Soto C. J. John O. P. Andersen S. C. (2021). Development and validation of the Danish big five Inventory-2: domain-and facet-level structure, construct validity, and reliability. Eur. J. Psychol. Assess. 37, 42–51. doi: 10.1027/1015-5759/a000570

[ref66] Wada S. (1996). Construction of the big five scales of personality trait terms and concurrent validity with NPI. Jpn. J. Psychol. 67, 61–67. doi: 10.4992/jjpsy.67.61

[ref67] Weisberg Y. J. DeYoung C. G. Hirsh J. B. (2011). Gender differences in personality across the ten aspects of the big five. Front. Psychol. 2:178. doi: 10.3389/fpsyg.2011.00178, PMID: 21866227 PMC3149680

[ref68] Zhang B. Li Y. M. Li J. Luo J. Ye Y. Yin L.. (2021). The big five Inventory-2 in China: a comprehensive psychometric evaluation in four diverse samples. Assessment 29, 1262–1284. doi: 10.1177/10731911211008245, PMID: 33884926

