# Supplementary material for: A validation of the Japanese adaptation of the Big Five Inventory-2
Source: Front Psychol. 2022 Oct 14;13:924351. doi: 10.3389/fpsyg.2022.924351 (PMC9614413; doi:10.3389/fpsyg.2022.924351)
Supplement: Supplementary file 1 [file Table_1.docx]

Supplementary Material

# Table S1: Japanese version of the items

**Japanese instruction**: 以下の項目は，あなたにどの程度あてはまりますか。最も近いと思う数字を選んでください。

1:全くあてはまらない, 2:あてはまらない, 3:どちらともいえない, 4:あてはまる, 5:とてもよくあてはまる

|  | English | Japanese | Domain | Facet |  |
| --- | --- | --- | --- | --- | --- |
| 1 | Is outgoing, sociable | 積極的で，社交的である | Extraversion | Sociability |  |
| 2 | Is compassionate, has a soft heart | 思いやりがあり，優しい | Agreeableness | Compassion |  |
| 3 | Tends to be disorganized | 行き当たりばったりな方だ | Conscientiousness | Organization | * |
| 4 | Is relaxed, handles stress well | リラックスしていて，ストレスにうまく対処している | Negative Emotionality | Anxiety | * |
| 5 | Has few artistic interests | 芸術的関心があまりない | Open-Mindedness | Aesthetic Sensitivity | * |
| 6 | Has an assertive personality | 積極的な性格だ | Extraversion | Assertiveness |  |
| 7 | Is respectful, treats others with respect | 礼儀正しく，他人に敬意をもって接する | Agreeableness | Respectful |  |
| 8 | Tends to be lazy | だらだらと過ごす方だ | Conscientiousness | Productiveness | * |
| 9 | Stays optimistic after experiencing a setback | 失敗を経験しても楽天的なままでいる | Negative Emotionality | Depression | * |
| 10 | Is curious about many different things | 色々な物事に対する好奇心が強い | Open-Mindedness | Intellectual Curiosity |  |
| 11 | Rarely feels excited or eager | めったに興奮したり，熱狂したりしない | Extraversion | Energy Level | * |
| 12 | Tends to find fault with others | 他人の欠点を見つけ出す方だ | Agreeableness | Trust | * |
| 13 | Is dependable, steady | しっかりしていて，真面目だ | Conscientiousness | Responsibility |  |
| 14 | Is moody, has up and down mood swings | 不機嫌になりやすく，感情の起伏が激しい | Negative Emotionality | Emotional Volatility |  |
| 15 | Is inventive, finds clever ways to do things | 創意工夫が得意で，うまい方法を思いつくことができる | Open-Mindedness | Creative Imagination |  |
| 16 | Tends to be quiet | 無口な方だ | Extraversion | Sociability | * |
| 17 | Feels little sympathy for others | 他人のことを思って心が痛むことはほとんどない | Agreeableness | Compassion | * |
| 18 | Is systematic, likes to keep things in order | 几帳面で，規則正しく整えることが好きだ | Conscientiousness | Organization |  |
| 19 | Can be tense | 神経が張り詰めることがある | Negative Emotionality | Anxiety |  |
| 20 | Is fascinated by art, music, or literature | 芸術，音楽，文学に魅了されている | Open-Mindedness | Aesthetic Sensitivity |  |
| 21 | Is dominant, acts as a leader | 上に立つ方で，リーダーとして活動する | Extraversion | Assertiveness |  |
| 22 | Starts arguments with others | 他人と言い争いを始める | Agreeableness | Respectful | * |
| 23 | Has difficulty getting started on tasks | なかなか作業に取り掛かることができない | Conscientiousness | Productiveness | * |
| 24 | Feels secure, comfortable with self | 安心感を抱いており，心地よい | Negative Emotionality | Depression | * |
| 25 | Avoids intellectual, philosophical discussions | 知的で哲学的な考察を避けるようにしている | Open-Mindedness | Intellectual Curiosity | * |
| 26 | Is less active than other people | 他の人と比べて活発ではない | Extraversion | Energy Level | * |
| 27 | Has a forgiving nature | 他人を大目に見る寛大な人間だ | Agreeableness | Trust |  |
| 28 | Can be somewhat careless | 少し不注意なところがある | Conscientiousness | Responsibility | * |
| 29 | Is emotionally stable, not easily upset | 情緒が安定しており，簡単には取り乱さない | Negative Emotionality | Emotional Volatility | * |
| 30 | Has little creativity | 創造性がほとんどない | Open-Mindedness | Creative Imagination | * |
| 31 | Is sometimes shy, introverted | 内気なところがあり，内向的である | Extraversion | Sociability | * |
| 32 | Is helpful and unselfish with others | 進んで手伝おうとし，他人の利益を優先する | Agreeableness | Compassion |  |
| 33 | Keeps things neat and tidy | 物事をきれいに揃えたりまとめたりする | Conscientiousness | Organization |  |
| 34 | Worries a lot | 多くの悩みごとを抱えている | Negative Emotionality | Anxiety |  |
| 35 | Values art and beauty | 芸術と美を重視する | Open-Mindedness | Aesthetic Sensitivity |  |
| 36 | Finds it hard to influence people | 人々の行動を左右するような影響力をもつことは難しいと感じる | Extraversion | Assertiveness | * |
| 37 | Is sometimes rude to others | 他人を見下すことがある | Agreeableness | Respectful | * |
| 38 | Is efficient, gets things done | 手際よく行動し，物事を最後までやり遂げる | Conscientiousness | Productiveness |  |
| 39 | Often feels sad | よく悲しい気分になる | Negative Emotionality | Depression |  |
| 40 | Is complex, a deep thinker | 考え方が複雑で，深く考える人間だ | Open-Mindedness | Intellectual Curiosity |  |
| 41 | Is full of energy | 活力にあふれている | Extraversion | Energy Level |  |
| 42 | Is suspicious of others’ intentions | 他人が考えていることを怪しんで不信感を抱く | Agreeableness | Trust | * |
| 43 | Is reliable, can always be counted on | ちゃんとしていて，いつも周りから当てにされる | Conscientiousness | Responsibility |  |
| 44 | Keeps their emotions under control | 自分の感情をコントロールしている | Negative Emotionality | Emotional Volatility | * |
| 45 | Has difficulty imagining things | ものごとを自由に心に思い描くのは難しい | Open-Mindedness | Creative Imagination | * |
| 46 | Is talkative | おしゃべりな方だ | Extraversion | Sociability |  |
| 47 | Can be cold and uncaring | 冷淡で思いやりに欠けることがある | Agreeableness | Compassion | * |
| 48 | Leaves a mess, doesn’t clean up | 乱雑なものはそのままにして，きれいにしない | Conscientiousness | Organization | * |
| 49 | Rarely feels anxious or afraid | 不安や恐れを感じることはめったにない | Negative Emotionality | Anxiety | * |
| 50 | Thinks poetry and plays are boring | 詩や演劇をつまらないと思う | Open-Mindedness | Aesthetic Sensitivity | * |
| 51 | Prefers to have others take charge | 他の人にリーダーシップを発揮してもらうほうが良いと思う | Extraversion | Assertiveness | * |
| 52 | Is polite, courteous to others | 他人に丁寧で，礼儀正しい | Agreeableness | Respectful |  |
| 53 | Is persistent, works until the task is finished | 根気強く，与えられた課題が終わるまで取り組む | Conscientiousness | Productiveness |  |
| 54 | Tends to feel depressed, blue | 憂うつになり，落胆する方だ | Negative Emotionality | Depression |  |
| 55 | Has little interest in abstract ideas | 抽象的な知識にはほとんど関心がない | Open-Mindedness | Intellectual Curiosity | * |
| 56 | Shows a lot of enthusiasm | 情熱を大いに表に出す | Extraversion | Energy Level |  |
| 57 | Assumes the best about people | 人々のいちばん良いところを思い浮かべる | Agreeableness | Trust |  |
| 58 | Sometimes behaves irresponsibly | 無責任な行動をしてしまうことがある | Conscientiousness | Responsibility | * |
| 59 | Is temperamental, gets emotional easily | 神経質で，感情的になりやすい | Negative Emotionality | Emotional Volatility |  |
| 60 | Is original, comes up with new ideas | 個性的で，新しいアイディアを思いつく | Open-Mindedness | Creative Imagination |  |
| *Note.* The asterisk showed the reverse-keyed item. Extraversion=外向性, Sociability=社交性, Assertiveness=自己主張性, Energy Level=活力度, Agreeableness=協調性, Compassion=思いやり, Respectfulness=敬意, Trust=信用, Conscientiousness=勤勉性, Organization=秩序, Productiveness=生産性, Responsibility=責任感, Negative Emotionality=ネガティブ情動性, Anxiety=不安, Depression=抑うつ, Emotional Volatility=情緒不安定性, Open-Mindedness=開放性, Intellectual Curiosity=知的好奇心, Aesthetic Sensitivity=美的感性, Creative Imagination=創造的想像力 | | | | | |

# Table S2: Loadings from a Principal Components Analysis of the 60 Items (oblimin rotation)

|  |  |  | Student | | | | |  | Community | | | | |
| --- | --- | --- | --- | --- | --- | --- | --- | --- | --- | --- | --- | --- | --- |
|  |  | items | E | A | C | N | O |  | E | A | C | N | O |
| Extraversion | |  |  |  |  |  |  |  |  |  |  |  |  |
|  | Sociability |  |  |  |  |  |  |  |  |  |  |  |  |
|  |  | item 01 | **.76** | .14 | -.05 | .13 | .00 |  | **.70** | .14 | .05 | .23 | -.03 |
|  |  | item 16 | **-.78** | -.05 | .11 | .08 | .01 |  | **-.78** | -.11 | .08 | .14 | -.01 |
|  |  | item 31 | **-.81** | .06 | -.03 | -.06 | .00 |  | **-.70** | .02 | -.11 | -.20 | .05 |
|  |  | item 46 | **.68** | -.02 | -.11 | -.02 | -.08 |  | **.75** | .09 | -.21 | -.05 | -.09 |
|  | Assertiveness |  |  |  |  |  |  |  |  |  |  |  |  |
|  |  | item 06 | **.79** | -.01 | .01 | .09 | .05 |  | **.65** | .03 | .10 | .22 | .01 |
|  |  | item 21 | **.66** | -.13 | .21 | .02 | .01 |  | **.39** | -.13 | **.38** | .15 | .02 |
|  |  | item 36 | **-.37** | .27 | -.20 | -.19 | -.18 |  | **-.28** | .17 | -.21 | -.13 | -.21 |
|  |  | item 51 | **-.57** | .22 | -.28 | .02 | -.08 |  | **-.39** | .30 | **-.39** | -.03 | -.11 |
|  | Energy Level |  |  |  |  |  |  |  |  |  |  |  |  |
|  |  | item 11 | **-.51** | -.10 | .29 | .25 | -.06 |  | **-.56** | .17 | .20 | **.31** | -.10 |
|  |  | item 26 | **-.78** | -.07 | -.09 | .00 | -.06 |  | **-.64** | .03 | -.22 | -.21 | .00 |
|  |  | item 41 | **.68** | .13 | .06 | .12 | .01 |  | **.47** | -.01 | .13 | **.34** | .10 |
|  |  | item 56 | **.63** | .14 | -.17 | -.17 | .01 |  | **.58** | -.05 | -.09 | .10 | .10 |
|  |  |  |  |  |  |  |  |  |  |  |  |  |  |
| Agreeableness | |  |  |  |  |  |  |  |  |  |  |  |  |
|  | Compassion |  |  |  |  |  |  |  |  |  |  |  |  |
|  |  | item 02 | .18 | **.52** | .11 | .15 | .10 |  | .24 | **.57** | .18 | -.02 | .07 |
|  |  | item 17 | -.29 | **-.38** | -.02 | .29 | -.18 |  | **-.31** | **-.45** | -.01 | .28 | -.22 |
|  |  | item 32 | .03 | **.48** | .13 | .00 | .01 |  | .17 | **.35** | .00 | .14 | .08 |
|  |  | item 47 | -.19 | **-.61** | -.01 | .00 | .06 |  | -.29 | **-.52** | -.09 | .06 | -.17 |
|  | Respectfulness |  |  |  |  |  |  |  |  |  |  |  |  |
|  |  | item 07 | .06 | **.57** | .18 | -.02 | .07 |  | .17 | **.59** | .11 | -.05 | .10 |
|  |  | item 22 | **.33** | **-.41** | -.05 | **-.33** | -.04 |  | .19 | **-.55** | -.05 | -.06 | .08 |
|  |  | item 37 | -.04 | **-.47** | -.05 | -.20 | .16 |  | .03 | **-.57** | -.07 | -.20 | -.06 |
|  |  | item 52 | -.05 | **.58** | .13 | -.04 | .03 |  | .04 | **.59** | .28 | -.08 | .07 |
|  | Trust |  |  |  |  |  |  |  |  |  |  |  |  |
|  |  | item 12 | .12 | **-.35** | .15 | **-.33** | -.03 |  | .06 | **-.38** | .17 | **-.37** | -.02 |
|  |  | item 27 | -.08 | **.39** | -.06 | **.40** | .06 |  | -.15 | **.51** | -.15 | .29 | .14 |
|  |  | item 42 | -.14 | **-.33** | -.01 | **-.32** | .05 |  | -.04 | **-.32** | .19 | **-.45** | -.14 |
|  |  | item 57 | .23 | **.42** | -.16 | .17 | .09 |  | .21 | **.33** | .02 | .20 | .11 |
|  |  |  |  |  |  |  |  |  |  |  |  |  |  |
| Conscientiousness | |  |  |  |  |  |  |  |  |  |  |  |  |
|  | Organization |  |  |  |  |  |  |  |  |  |  |  |  |
|  |  | item 03 | .17 | -.04 | **-.62** | .09 | .03 |  | .09 | -.02 | **-.65** | .14 | .03 |
|  |  | item 18 | -.19 | -.08 | **.64** | .03 | .02 |  | -.14 | .06 | **.70** | -.11 | -.03 |
|  |  | item 33 | -.09 | -.02 | **.63** | .00 | .12 |  | -.07 | .02 | **.63** | -.03 | .05 |
|  |  | item 48 | .02 | -.07 | **-.60** | -.01 | -.10 |  | .03 | -.14 | **-.63** | .05 | -.02 |
|  | Productiveness |  |  |  |  |  |  |  |  |  |  |  |  |
|  |  | item 08 | -.16 | -.09 | **-.50** | .11 | -.08 |  | -.21 | .03 | **-.57** | -.03 | .02 |
|  |  | item 23 | -.21 | -.01 | **-.56** | -.07 | .04 |  | -.08 | -.11 | **-.52** | -.21 | .14 |
|  |  | item 38 | .18 | -.01 | **.53** | .17 | -.06 |  | .11 | .07 | **.55** | .09 | .09 |
|  |  | item 53 | .10 | **.35** | **.37** | .05 | -.13 |  | .05 | **.33** | **.49** | .03 | -.02 |
|  | Responsibility |  |  |  |  |  |  |  |  |  |  |  |  |
|  |  | item 13 | .02 | .14 | **.64** | -.09 | -.06 |  | .11 | **.31** | **.51** | -.14 | -.04 |
|  |  | item 28 | .02 | .07 | **-.55** | -.02 | .12 |  | .08 | .02 | **-.59** | -.19 | .08 |
|  |  | item 43 | **.34** | .08 | **.55** | .01 | -.08 |  | .26 | .09 | **.44** | .06 | .05 |
|  |  | item 58 | -.01 | -.17 | **-.56** | .03 | .11 |  | -.06 | -.29 | **-.49** | -.06 | -.06 |
|  |  |  |  |  |  |  |  |  |  |  |  |  |  |
| Negative Emotionality | |  |  |  |  |  |  |  |  |  |  |  |  |
|  | Anxiety |  |  |  |  |  |  |  |  |  |  |  |  |
|  |  | item 04 | .01 | .06 | -.07 | **.77** | .01 |  | .12 | .00 | .06 | **.69** | .11 |
|  |  | item 19 | -.14 | .02 | .21 | **-.62** | .03 |  | .03 | .08 | .16 | **-.72** | .06 |
|  |  | item 34 | -.09 | .04 | .08 | **-.76** | .01 |  | -.15 | .02 | .03 | **-.73** | .00 |
|  |  | item 49 | .07 | -.16 | -.01 | **.61** | -.15 |  | .04 | -.17 | .02 | **.75** | -.03 |
|  | Depression |  |  |  |  |  |  |  |  |  |  |  |  |
|  |  | item 09 | .02 | -.14 | **-.31** | **.56** | .09 |  | .08 | -.10 | -.25 | **.67** | .10 |
|  |  | item 24 | .13 | .18 | -.08 | **.57** | .00 |  | .13 | .14 | -.04 | **.59** | .13 |
|  |  | item 39 | -.13 | .02 | -.01 | **-.71** | .10 |  | -.13 | -.03 | -.07 | **-.69** | .07 |
|  |  | item 54 | -.23 | .07 | -.06 | **-.72** | .09 |  | -.19 | -.01 | -.15 | **-.67** | .01 |
|  | Emotional Volatility |  |  |  |  |  |  |  |  |  |  |  |  |
|  |  | item 14 | .14 | -.17 | -.15 | **-.61** | -.09 |  | .06 | **-.39** | -.15 | **-.46** | -.06 |
|  |  | item 29 | -.16 | -.03 | .18 | **.74** | -.03 |  | -.12 | .19 | .27 | **.59** | -.04 |
|  |  | item 44 | -.13 | .18 | .23 | **.61** | .00 |  | -.15 | .30 | **.31** | **.41** | -.01 |
|  |  | item 59 | .12 | -.14 | -.02 | **-.71** | -.11 |  | .14 | -.23 | -.17 | **-.57** | -.13 |
|  |  |  |  |  |  |  |  |  |  |  |  |  |  |
| Open-Mindedness | |  |  |  |  |  |  |  |  |  |  |  |  |
|  | Intellectual Curiosity |  |  |  |  |  |  |  |  |  |  |  |  |
|  |  | item 10 | **.34** | .02 | .02 | .08 | **.47** |  | .27 | -.02 | .19 | .13 | **.32** |
|  |  | item 25 | .03 | -.03 | -.13 | .16 | **-.47** |  | -.07 | .11 | -.25 | .18 | **-.31** |
|  |  | item 40 | -.01 | .01 | .21 | **-.43** | **.32** |  | -.07 | -.13 | .28 | **-.48** | .23 |
|  |  | item 55 | -.02 | .04 | -.02 | .09 | **-.54** |  | .00 | -.09 | -.08 | -.03 | **-.54** |
|  | Aesthetic Sensitivity |  |  |  |  |  |  |  |  |  |  |  |  |
|  |  | item 05 | .11 | -.08 | .05 | .08 | **-.77** |  | .07 | -.01 | .06 | .04 | **-.78** |
|  |  | item 20 | -.14 | .08 | -.07 | -.02 | **.70** |  | -.07 | .05 | -.11 | -.02 | **.84** |
|  |  | item 35 | -.12 | .04 | -.05 | -.06 | **.71** |  | -.14 | .03 | -.08 | .03 | **.78** |
|  |  | item 50 | -.05 | -.18 | .04 | .12 | **-.54** |  | -.11 | -.23 | .06 | .14 | **-.61** |
|  | Creative Imagination |  |  |  |  |  |  |  |  |  |  |  |  |
|  |  | item 15 | .14 | -.17 | .05 | .16 | **.61** |  | .13 | -.23 | **.44** | .24 | **.35** |
|  |  | item 30 | -.23 | .09 | -.05 | -.07 | **-.61** |  | -.14 | .11 | **-.36** | -.10 | **-.51** |
|  |  | item 45 | -.11 | -.12 | .14 | -.05 | -.22 |  | -.16 | .00 | -.15 | -.14 | **-.37** |
|  |  | item 60 | .26 | -.19 | .00 | .11 | **.63** |  | .19 | -.30 | .27 | .17 | **.46** |
|  |  | |  |  |  |  |  |  |  |  |  |  |  |
|  | Correlations between components | |  |  |  |  |  |  |  |  |  |  |  |
|  |  | A | .04 |  |  |  |  |  | .05 |  |  |  |  |
|  |  | C | .04 | .12 |  |  |  |  | .24 | .19 |  |  |  |
|  |  | N | .14 | .14 | .07 |  |  |  | .28 | .16 | .24 |  |  |
|  |  | O | .14 | .05 | -.02 | -.11 |  |  | .23 | .15 | .19 | .11 |  |
| *Note*. Each individual item’s response was subtracted from the within-person mean. Absolute loadings of .30 or stronger are bolded. Cumulative proportion of variance is .44 (student sample) and .46 (community sample). | | | | | | | | | | | | | |

# Table S3: Loadings from an Exploratory Factor Analysis of the 60 Items (varimax rotation)

|  |  |  | Student | | | | |  | Community | | | | |
| --- | --- | --- | --- | --- | --- | --- | --- | --- | --- | --- | --- | --- | --- |
|  |  | items | E | A | C | N | O |  | E | A | C | N | O |
| Extraversion | |  |  |  |  |  |  |  |  |  |  |  |  |
|  | Sociability |  |  |  |  |  |  |  |  |  |  |  |  |
|  |  | item 01 | **.77** | .21 | -.02 | .19 | .06 |  | **.72** | .24 | .17 | **.31** | .02 |
|  |  | item 16 | **-.74** | -.08 | .09 | .00 | -.07 |  | **-.68** | -.14 | -.02 | .02 | -.07 |
|  |  | item 31 | **-.81** | -.01 | -.06 | -.12 | -.05 |  | **-.71** | -.08 | -.21 | -.28 | -.02 |
|  |  | item 46 | **.64** | .01 | -.10 | .05 | .00 |  | **.62** | .09 | -.11 | .07 | .00 |
|  | Assertiveness |  |  |  |  |  |  |  |  |  |  |  |  |
|  |  | item 06 | **.80** | .06 | .04 | .14 | .10 |  | **.67** | .14 | .21 | **.30** | .05 |
|  |  | item 21 | **.62** | -.07 | .22 | .07 | .06 |  | **.43** | .00 | **.42** | .20 | .07 |
|  |  | item 36 | **-.37** | .18 | -.19 | -.16 | -.15 |  | **-.33** | .05 | -.27 | -.17 | -.19 |
|  |  | item 51 | **-.54** | .16 | -.27 | -.01 | -.11 |  | **-.43** | .16 | **-.41** | -.08 | -.12 |
|  | Energy Level |  |  |  |  |  |  |  |  |  |  |  |  |
|  |  | item 11 | **-.44** | -.06 | .25 | .18 | -.13 |  | **-.42** | .17 | .14 | .21 | -.12 |
|  |  | item 26 | **-.78** | -.13 | -.11 | -.07 | -.12 |  | **-.67** | -.08 | **-.33** | -.30 | -.06 |
|  |  | item 41 | **.65** | .18 | .10 | .18 | .07 |  | **.52** | .09 | .26 | **.40** | .15 |
|  |  | item 56 | **.54** | .11 | -.14 | -.09 | .10 |  | **.52** | .00 | .04 | .17 | .13 |
|  |  |  |  |  |  |  |  |  |  |  |  |  |  |
| Agreeableness | |  |  |  |  |  |  |  |  |  |  |  |  |
|  | Compassion |  |  |  |  |  |  |  |  |  |  |  |  |
|  |  | item 02 | .19 | **.50** | .15 | .18 | .10 |  | .22 | **.59** | .26 | .07 | .10 |
|  |  | item 17 | -.24 | **-.32** | -.03 | .22 | -.22 |  | -.24 | **-.42** | -.09 | .16 | -.21 |
|  |  | item 32 | .03 | **.40** | .16 | .04 | .02 |  | .13 | **.32** | .11 | .18 | .09 |
|  |  | item 47 | -.15 | **-.53** | -.06 | -.07 | .01 |  | -.26 | **-.54** | -.19 | -.03 | -.19 |
|  | Respectfulness |  |  |  |  |  |  |  |  |  |  |  |  |
|  |  | item 07 | .06 | **.52** | .20 | .02 | .08 |  | .14 | **.59** | .18 | .04 | .14 |
|  |  | item 22 | .27 | **-.41** | -.08 | -.29 | .00 |  | .18 | **-.47** | -.06 | -.07 | .06 |
|  |  | item 37 | -.01 | **-.40** | -.11 | -.24 | .11 |  | .02 | **-.53** | -.16 | -.22 | -.08 |
|  |  | item 52 | -.05 | **.52** | .15 | .01 | .04 |  | .03 | **.60** | **.31** | .00 | .11 |
|  | Trust |  |  |  |  |  |  |  |  |  |  |  |  |
|  |  | item 12 | .09 | **-.29** | .09 | **-.30** | -.01 |  | .04 | **-.34** | .05 | **-.33** | -.04 |
|  |  | item 27 | -.05 | **.37** | -.01 | **.37** | .01 |  | -.11 | **.46** | -.03 | .27 | .10 |
|  |  | item 42 | -.13 | **-.30** | -.06 | **-.33** | .03 |  | -.08 | **-.32** | .04 | **-.43** | -.13 |
|  |  | item 57 | .20 | **.35** | -.08 | .20 | .10 |  | .21 | **.33** | .14 | .24 | .13 |
|  |  |  |  |  |  |  |  |  |  |  |  |  |  |
| Conscientiousness | |  |  |  |  |  |  |  |  |  |  |  |  |
|  | Organization |  |  |  |  |  |  |  |  |  |  |  |  |
|  |  | item 03 | .15 | -.09 | **-.56** | .10 | .03 |  | .03 | -.08 | **-.55** | .08 | -.03 |
|  |  | item 18 | -.16 | -.03 | **.58** | .01 | .00 |  | -.07 | .13 | **.59** | -.07 | .01 |
|  |  | item 33 | -.06 | .03 | **.58** | -.01 | .10 |  | .02 | .10 | **.56** | .01 | .07 |
|  |  | item 48 | .00 | -.11 | **-.56** | -.01 | -.08 |  | -.04 | -.22 | **-.57** | .00 | -.06 |
|  | Productiveness |  |  |  |  |  |  |  |  |  |  |  |  |
|  |  | item 08 | -.15 | -.12 | **-.46** | .09 | -.10 |  | -.25 | -.06 | **-.55** | -.10 | -.05 |
|  |  | item 23 | -.19 | -.06 | **-.53** | -.09 | .02 |  | -.13 | -.18 | **-.50** | -.24 | .04 |
|  |  | item 38 | .18 | .06 | **.51** | .18 | -.06 |  | .17 | .16 | **.56** | .15 | .13 |
|  |  | item 53 | .08 | **.34** | **.37** | .09 | -.09 |  | .07 | **.36** | **.50** | .10 | .03 |
|  | Responsibility |  |  |  |  |  |  |  |  |  |  |  |  |
|  |  | item 13 | .01 | .18 | **.60** | -.07 | -.03 |  | .10 | **.34** | **.47** | -.05 | .03 |
|  |  | item 28 | .03 | .03 | **-.50** | -.03 | .10 |  | -.01 | -.08 | **-.53** | -.19 | .02 |
|  |  | item 43 | **.30** | .12 | **.54** | .06 | -.03 |  | .27 | .17 | **.47** | .14 | .10 |
|  |  | item 58 | .01 | -.18 | **-.52** | .00 | .08 |  | -.11 | **-.37** | **-.50** | -.13 | -.09 |
|  |  |  |  |  |  |  |  |  |  |  |  |  |  |
| Negative Emotionality | |  |  |  |  |  |  |  |  |  |  |  |  |
|  | Anxiety |  |  |  |  |  |  |  |  |  |  |  |  |
|  |  | item 04 | .05 | .12 | -.02 | **.76** | -.03 |  | .23 | .11 | .18 | **.67** | .12 |
|  |  | item 19 | -.16 | -.04 | .16 | **-.59** | .03 |  | -.06 | .01 | .05 | **-.64** | .07 |
|  |  | item 34 | -.13 | -.03 | .03 | **-.74** | .05 |  | -.23 | -.07 | -.10 | **-.72** | -.04 |
|  |  | item 49 | .09 | -.09 | .02 | **.57** | -.18 |  | .14 | -.08 | .11 | **.70** | .00 |
|  | Depression |  |  |  |  |  |  |  |  |  |  |  |  |
|  |  | item 09 | .06 | -.09 | -.27 | **.50** | .04 |  | .17 | -.03 | -.11 | **.60** | .05 |
|  |  | item 24 | .14 | .22 | -.02 | **.56** | -.02 |  | .20 | .21 | .10 | **.58** | .14 |
|  |  | item 39 | -.16 | -.05 | -.05 | **-.71** | .11 |  | -.20 | -.11 | -.19 | **-.68** | .01 |
|  |  | item 54 | -.25 | -.01 | -.11 | **-.72** | .10 |  | -.28 | -.11 | -.27 | **-.69** | -.03 |
|  | Emotional Volatility |  |  |  |  |  |  |  |  |  |  |  |  |
|  |  | item 14 | .07 | -.24 | -.18 | **-.55** | -.03 |  | -.02 | **-.44** | -.24 | **-.45** | -.07 |
|  |  | item 29 | -.10 | .07 | .22 | **.70** | -.09 |  | .00 | .28 | **.33** | **.57** | -.03 |
|  |  | item 44 | -.07 | .26 | .26 | **.58** | -.05 |  | -.06 | **.35** | **.35** | **.41** | .02 |
|  |  | item 59 | .05 | -.23 | -.06 | **-.65** | -.04 |  | .02 | **-.31** | -.27 | **-.54** | -.13 |
|  |  |  |  |  |  |  |  |  |  |  |  |  |  |
| Open-Mindedness | |  |  |  |  |  |  |  |  |  |  |  |  |
|  | Intellectual Curiosity |  |  |  |  |  |  |  |  |  |  |  |  |
|  |  | item 10 | **.36** | .06 | .02 | .07 | **.43** |  | **.35** | .10 | .28 | .17 | **.27** |
|  |  | item 25 | -.02 | -.04 | -.09 | .18 | **-.37** |  | -.13 | .02 | -.23 | .12 | **-.23** |
|  |  | item 40 | .01 | -.01 | .16 | **-.42** | **.27** |  | -.05 | -.10 | .19 | **-.42** | .14 |
|  |  | item 55 | -.07 | .02 | .01 | .12 | **-.44** |  | -.09 | -.17 | -.16 | -.07 | **-.43** |
|  | Aesthetic Sensitivity |  |  |  |  |  |  |  |  |  |  |  |  |
|  |  | item 05 | .06 | -.09 | .07 | .13 | **-.78** |  | -.04 | -.10 | -.01 | .01 | **-.74** |
|  |  | item 20 | -.08 | .09 | -.09 | -.06 | **.70** |  | .03 | .13 | -.03 | .01 | **.81** |
|  |  | item 35 | -.07 | .04 | -.07 | -.11 | **.71** |  | -.02 | .11 | -.01 | .04 | **.74** |
|  |  | item 50 | -.09 | -.18 | .04 | .13 | **-.52** |  | -.15 | -.27 | -.04 | .07 | **-.56** |
|  | Creative Imagination |  |  |  |  |  |  |  |  |  |  |  |  |
|  |  | item 15 | .19 | -.13 | .05 | .10 | **.53** |  | .26 | -.08 | **.51** | .26 | **.32** |
|  |  | item 30 | -.26 | .06 | -.05 | -.03 | **-.55** |  | -.28 | -.03 | **-.44** | -.15 | **-.47** |
|  |  | item 45 | -.11 | -.08 | .10 | -.04 | -.18 |  | -.24 | -.12 | -.23 | -.17 | **-.32** |
|  |  | item 60 | **.30** | -.15 | .00 | .06 | **.57** |  | **.32** | -.15 | **.36** | .19 | **.42** |
| *Note*. Each individual item’s response was subtracted from the within-person mean. Absolute loadings of .30 or stronger are bolded. Cumulative proportion of variance is .39 (student sample) and .42 (community sample). | | | | | | | | | | | | | |

# Table S4: Loadings from an Exploratory Factor Analysis of the 60 Items (oblimin rotation)

|  |  |  | Student | | | | |  | Community | | | | |
| --- | --- | --- | --- | --- | --- | --- | --- | --- | --- | --- | --- | --- | --- |
|  |  | items | E | A | C | N | O |  | E | A | C | N | O |
| Extraversion | |  |  |  |  |  |  |  |  |  |  |  |  |
|  | Sociability |  |  |  |  |  |  |  |  |  |  |  |  |
|  |  | item 01 | **.79** | .14 | -.05 | .11 | -.02 |  | **.74** | .15 | .01 | .18 | -.05 |
|  |  | item 16 | **-.77** | -.04 | .11 | .08 | .01 |  | **-.74** | -.09 | .08 | .15 | -.02 |
|  |  | item 31 | **-.81** | .07 | -.03 | -.05 | .02 |  | **-.71** | .02 | -.09 | -.16 | .05 |
|  |  | item 46 | **.66** | -.03 | -.12 | -.02 | -.07 |  | **.68** | .05 | -.22 | -.04 | -.03 |
|  | Assertiveness |  |  |  |  |  |  |  |  |  |  |  |  |
|  |  | item 06 | **.80** | -.02 | .01 | .06 | .02 |  | **.67** | .04 | .08 | .18 | -.01 |
|  |  | item 21 | **.60** | -.15 | .21 | .02 | .01 |  | **.37** | -.10 | .37 | .12 | -.01 |
|  |  | item 36 | **-.32** | .25 | -.19 | -.16 | -.13 |  | **-.26** | .14 | -.22 | -.11 | -.15 |
|  |  | item 51 | **-.51** | .23 | -.27 | .03 | -.06 |  | **-.37** | .25 | -.40 | .00 | -.05 |
|  | Energy Level |  |  |  |  |  |  |  |  |  |  |  |  |
|  |  | item 11 | **-.47** | -.06 | .25 | .22 | -.08 |  | **-.47** | .19 | .15 | .29 | -.11 |
|  |  | item 26 | **-.80** | -.06 | -.09 | .01 | -.04 |  | **-.64** | .03 | -.21 | -.17 | .02 |
|  |  | item 41 | **.66** | .10 | .07 | .12 | .01 |  | **.46** | -.02 | .14 | **.32** | .09 |
|  |  | item 56 | **.57** | .09 | -.15 | -.14 | .04 |  | **.52** | -.07 | -.05 | .08 | .10 |
|  |  |  |  |  |  |  |  |  |  |  |  |  |  |
| Agreeableness | |  |  |  |  |  |  |  |  |  |  |  |  |
|  | Compassion |  |  |  |  |  |  |  |  |  |  |  |  |
|  |  | item 02 | .21 | **.46** | .12 | .15 | .08 |  | .24 | **.55** | .15 | -.01 | .06 |
|  |  | item 17 | -.29 | **-.31** | -.03 | .25 | -.17 |  | -.28 | **-.40** | -.02 | .24 | -.19 |
|  |  | item 32 | .06 | **.39** | .13 | .02 | .01 |  | .12 | **.28** | .02 | .15 | .07 |
|  |  | item 47 | -.20 | **-.51** | -.03 | -.03 | .04 |  | -.28 | **-.49** | -.08 | .06 | -.15 |
|  | Respectfulness |  |  |  |  |  |  |  |  |  |  |  |  |
|  |  | item 07 | .10 | **.50** | .17 | -.01 | .06 |  | .16 | **.56** | .07 | -.03 | .11 |
|  |  | item 22 | .26 | **-.40** | -.05 | **-.30** | -.04 |  | .15 | **-.49** | -.01 | -.07 | .06 |
|  |  | item 37 | -.04 | **-.38** | -.07 | -.21 | .11 |  | .03 | **-.50** | -.06 | -.20 | -.07 |
|  |  | item 52 | .00 | **.51** | .13 | -.02 | .03 |  | .04 | **.58** | .23 | -.06 | .08 |
|  | Trust |  |  |  |  |  |  |  |  |  |  |  |  |
|  |  | item 12 | .09 | -.27 | .12 | **-.31** | -.03 |  | .06 | **-.32** | .15 | -.34 | -.05 |
|  |  | item 27 | -.04 | **.34** | -.04 | **.37** | .04 |  | -.12 | **.45** | -.14 | .29 | .11 |
|  |  | item 42 | -.14 | -.26 | -.03 | **-.31** | .03 |  | -.05 | **-.27** | .17 | **-.43** | -.14 |
|  |  | item 57 | .22 | **.32** | -.11 | .17 | .09 |  | .19 | **.27** | .03 | .19 | .10 |
|  |  |  |  |  |  |  |  |  |  |  |  |  |  |
| Conscientiousness | |  |  |  |  |  |  |  |  |  |  |  |  |
|  | Organization |  |  |  |  |  |  |  |  |  |  |  |  |
|  |  | item 03 | .16 | -.06 | **-.57** | .10 | .03 |  | .09 | -.05 | **-.59** | .11 | .03 |
|  |  | item 18 | -.19 | -.07 | **.59** | .03 | .01 |  | -.13 | .10 | **.63** | -.10 | -.04 |
|  |  | item 33 | -.09 | -.02 | **.59** | .00 | .10 |  | -.04 | .04 | **.58** | -.04 | .02 |
|  |  | item 48 | .01 | -.06 | **-.56** | -.01 | -.07 |  | .01 | -.17 | **-.57** | .05 | .00 |
|  | Productiveness |  |  |  |  |  |  |  |  |  |  |  |  |
|  |  | item 08 | -.15 | -.07 | **-.46** | .11 | -.07 |  | -.19 | .02 | **-.53** | -.02 | .02 |
|  |  | item 23 | -.18 | .00 | **-.52** | -.07 | .04 |  | -.07 | -.11 | **-.46** | -.19 | .11 |
|  |  | item 38 | .16 | -.01 | **.49** | .15 | -.07 |  | .09 | .07 | **.53** | .09 | .06 |
|  |  | item 53 | .10 | .30 | **.35** | .06 | -.10 |  | .03 | .31 | **.46** | .04 | -.03 |
|  | Responsibility |  |  |  |  |  |  |  |  |  |  |  |  |
|  |  | item 13 | .02 | .13 | **.60** | -.09 | -.05 |  | .08 | .30 | **.45** | -.12 | -.02 |
|  |  | item 28 | .05 | .07 | **-.50** | -.02 | .10 |  | .08 | -.02 | **-.53** | -.17 | .08 |
|  |  | item 43 | **.30** | .05 | **.53** | .02 | -.06 |  | .22 | .09 | **.43** | .06 | .03 |
|  |  | item 58 | .00 | -.15 | **-.51** | .02 | .09 |  | -.06 | -.30 | **-.45** | -.07 | -.03 |
|  |  |  |  |  |  |  |  |  |  |  |  |  |  |
| Negative Emotionality | |  |  |  |  |  |  |  |  |  |  |  |  |
|  | Anxiety |  |  |  |  |  |  |  |  |  |  |  |  |
|  |  | item 04 | -.01 | .04 | -.07 | **.77** | .02 |  | .12 | .00 | .05 | **.66** | .09 |
|  |  | item 19 | -.12 | .02 | .20 | **-.59** | .01 |  | .01 | .07 | .15 | **-.68** | .07 |
|  |  | item 34 | -.07 | .05 | .07 | **-.74** | .01 |  | -.14 | .03 | .04 | **-.72** | -.01 |
|  |  | item 49 | .04 | -.15 | -.02 | **.57** | -.15 |  | .03 | -.17 | .02 | **.72** | -.02 |
|  | Depression |  |  |  |  |  |  |  |  |  |  |  |  |
|  |  | item 09 | .01 | -.12 | -.29 | **.52** | .07 |  | .11 | -.10 | -.21 | **.62** | .05 |
|  |  | item 24 | .12 | .15 | -.07 | **.55** | .00 |  | .12 | .11 | -.03 | **.56** | .12 |
|  |  | item 39 | -.11 | .04 | .00 | **-.70** | .08 |  | -.11 | -.02 | -.06 | **-.68** | .04 |
|  |  | item 54 | -.20 | .08 | -.06 | **-.71** | .08 |  | -.17 | .00 | -.14 | **-.66** | .01 |
|  | Emotional Volatility |  |  |  |  |  |  |  |  |  |  |  |  |
|  |  | item 14 | .10 | -.17 | -.15 | **-.56** | -.07 |  | .03 | **-.38** | -.12 | **-.44** | -.05 |
|  |  | item 29 | -.16 | -.01 | .18 | **.72** | -.04 |  | -.09 | .21 | .23 | **.57** | -.06 |
|  |  | item 44 | -.11 | .18 | .22 | **.58** | -.01 |  | -.13 | **.30** | .27 | **.41** | -.01 |
|  |  | item 59 | .09 | -.16 | -.02 | **-.66** | -.09 |  | .12 | -.23 | -.16 | **-.55** | -.11 |
|  |  |  |  |  |  |  |  |  |  |  |  |  |  |
| Open-Mindedness | |  |  |  |  |  |  |  |  |  |  |  |  |
|  | Intellectual Curiosity |  |  |  |  |  |  |  |  |  |  |  |  |
|  |  | item 10 | .34 | .00 | .03 | .08 | **.40** |  | .29 | .00 | .21 | .10 | **.23** |
|  |  | item 25 | -.02 | -.03 | -.12 | .16 | **-.36** |  | -.09 | .07 | -.24 | .16 | **-.21** |
|  |  | item 40 | .02 | .01 | .19 | -.41 | .24 |  | -.05 | -.09 | .28 | -.44 | **.13** |
|  |  | item 55 | -.05 | .04 | -.02 | .09 | **-.44** |  | -.02 | -.10 | -.11 | -.04 | **-.42** |
|  | Aesthetic Sensitivity |  |  |  |  |  |  |  |  |  |  |  |  |
|  |  | item 05 | .09 | -.07 | .04 | .06 | **-.79** |  | .05 | .00 | .02 | .03 | **-.77** |
|  |  | item 20 | -.11 | .07 | -.05 | .00 | **.71** |  | -.06 | .03 | -.08 | -.01 | **.84** |
|  |  | item 35 | -.10 | .02 | -.04 | -.04 | **.72** |  | -.12 | .01 | -.04 | .04 | **.77** |
|  |  | item 50 | -.09 | -.16 | .02 | .10 | **-.51** |  | -.11 | -.20 | .02 | .13 | **-.57** |
|  | Creative Imagination |  |  |  |  |  |  |  |  |  |  |  |  |
|  |  | item 15 | .14 | -.19 | .07 | .14 | **.54** |  | .13 | -.21 | **.48** | .21 | **.26** |
|  |  | item 30 | -.22 | .12 | -.07 | -.06 | **-.54** |  | -.16 | .10 | **-.40** | -.08 | **-.42** |
|  |  | item 45 | -.11 | -.07 | .11 | -.04 | -.18 |  | -.17 | -.02 | -.17 | -.12 | **-.29** |
|  |  | item 60 | .25 | -.21 | .02 | .09 | **.56** |  | .19 | -.28 | .33 | .14 | **.37** |
|  |  |  |  |  |  |  |  |  |  |  |  |  |  |
|  | Correlations between factors | |  |  |  |  |  |  |  |  |  |  |  |
|  |  | A | .00 |  |  |  |  |  | .06 |  |  |  |  |
|  |  | C | .07 | .14 |  |  |  |  | .30 | .24 |  |  |  |
|  |  | N | .17 | .15 | .09 |  |  |  | .35 | .18 | .27 |  |  |
|  |  | O | .14 | .06 | -.03 | -.13 |  |  | .23 | .15 | .20 | .10 |  |
| *Note*. Each individual item’s response was subtracted from the within-person mean. Absolute loadings of .30 or stronger are bolded. Cumulative proportion of variance is .39 (student sample) and .42 (community sample). | | | | | | | | | | | | | |

# Table S5: Factor loadings in the three facets plus acquiescence model

|  |  |  | Student | Community |  |  |  |  | Student | Community |
| --- | --- | --- | --- | --- | --- | --- | --- | --- | --- | --- |
| **Extraversion** | |  |  |  |  | **Negative Emotionality** | |  |  |  |
|  | Sociability |  |  |  |  |  | Anxiety |  |  |  |
|  |  | ~item 01 | .82 | .86 |  |  |  | ~item 04 | .79 | .73 |
|  |  | ~item 16 | -.75 | -.60 |  |  |  | ~item 19 | -.60 | -.55 |
|  |  | ~item 31 | -.82 | -.82 |  |  |  | ~item 34 | -.78 | -.76 |
|  |  | ~item 46 | .68 | .57 |  |  |  | ~item 49 | .55 | .66 |
|  | Assertiveness |  |  |  |  |  | Depression |  |  |  |
|  |  | ~item 06 | .87 | .82 |  |  |  | ~item 09 | .45 | .53 |
|  |  | ~item 21 | .63 | .60 |  |  |  | ~item 24 | .57 | .60 |
|  |  | ~item 36 | -.39 | -.38 |  |  |  | ~item 39 | -.76 | -.74 |
|  |  | ~item 51 | -.56 | -.53 |  |  |  | ~item 54 | -.78 | -.79 |
|  | Energy Level |  |  |  |  |  | Emotional Volatility |  |  |  |
|  |  | ~item 11 | .40 | .20 |  |  |  | ~item 14 | .72 | .72 |
|  |  | ~item 26 | .84 | .81 |  |  |  | ~item 29 | -.75 | -.77 |
|  |  | ~item 41 | -.73 | -.73 |  |  |  | ~item 44 | -.68 | -.61 |
|  |  | ~item 56 | -.54 | -.57 |  |  |  | ~item 59 | .76 | .75 |
|  |  |  |  |  |  |  |  |  |  |  |
|  | Sociability~Assertiveness |  | .91 | .91 |  |  | Anxiety~Depression |  | 1.00 | 1.00 |
|  | Sociability~Energy Level |  | -.90 | -.89 |  |  | Anxiety~Emotional Volatility |  | -.74 | -.70 |
|  | Assertiveness~Energy Level |  | -.89 | -.89 |  |  | Depression~Emotional Volatility |  | -.74 | -.72 |
|  |  |  |  |  |  |  |  |  |  |  |
| **Agreeableness** | |  |  |  |  | **Open-Mindedness** | |  |  |  |
|  | Compassion |  |  |  |  |  | Intellectual Curiosity |  |  |  |
|  |  | ~item 02 | .62 | .73 |  |  |  | ~item 10 | .56 | .63 |
|  |  | ~item 17 | -.34 | -.44 |  |  |  | ~item 25 | -.51 | -.31 |
|  |  | ~item 32 | .48 | .40 |  |  |  | ~item 40 | .45 | .20 |
|  |  | ~item 47 | -.64 | -.68 |  |  |  | ~item 55 | -.56 | -.47 |
|  | Respectfulness |  |  |  |  |  | Aesthetic Sensitivity |  |  |  |
|  |  | ~item 07 | .74 | .67 |  |  |  | ~item 05 | .82 | .75 |
|  |  | ~item 22 | -.36 | -.35 |  |  |  | ~item 20 | -.81 | -.85 |
|  |  | ~item 37 | -.31 | -.62 |  |  |  | ~item 35 | -.79 | -.76 |
|  |  | ~item 52 | .69 | .64 |  |  |  | ~item 50 | .54 | .57 |
|  | Trust |  |  |  |  |  | Creative Imagination |  |  |  |
|  |  | ~item 12 | .44 | .53 |  |  |  | ~item 15 | .76 | .78 |
|  |  | ~item 27 | -.57 | -.55 |  |  |  | ~item 30 | -.72 | -.76 |
|  |  | ~item 42 | .51 | .47 |  |  |  | ~item 45 | -.20 | -.39 |
|  |  | ~item 57 | -.55 | -.46 |  |  |  | ~item 60 | .83 | .78 |
|  |  |  |  |  |  |  |  |  |  |  |
|  | Compassion  ~Respectfulness |  | .65 | .86 |  |  | Intellectual Curiosity  ~Aesthetic Sensitivity |  | -.58 | -.55 |
|  | Compassion~Trust |  | -.65 | -.68 |  |  | Intellectual Curiosity  ~Creative Imagination |  | .58 | .81 |
|  | Respectfulness~Trust |  | -.40 | -.73 |  |  | Aesthetic Sensitivity  ~Creative Imagination |  | -.47 | -.45 |
|  |  |  |  |  |  |  |  |  |  |  |
| **Conscientiousness** | |  |  |  |  |  |  |  |  |  |
|  | Organization |  |  |  |  |  |  |  |  |  |
|  |  | ~item 03 | .41 | .44 |  |  |  |  |  |  |
|  |  | ~item 18 | -.76 | -.69 |  |  |  |  |  |  |
|  |  | ~item 33 | -.75 | -.71 |  |  |  |  |  |  |
|  |  | ~item 48 | .67 | .63 |  |  |  |  |  |  |
|  | Productiveness |  |  |  |  |  |  |  |  |  |
|  |  | ~item 08 | .43 | .53 |  |  |  |  |  |  |
|  |  | ~item 23 | .63 | .58 |  |  |  |  |  |  |
|  |  | ~item 38 | -.66 | -.71 |  |  |  |  |  |  |
|  |  | ~item 53 | -.60 | -.67 |  |  |  |  |  |  |
|  | Responsibility |  |  |  |  |  |  |  |  |  |
|  |  | ~item 13 | .69 | .61 |  |  |  |  |  |  |
|  |  | ~item 28 | -.43 | -.48 |  |  |  |  |  |  |
|  |  | ~item 43 | .64 | .60 |  |  |  |  |  |  |
|  |  | ~item 58 | -.50 | -.54 |  |  |  |  |  |  |
|  |  |  |  |  |  |  |  |  |  |  |
|  | Organization  ~Productiveness |  | .51 | .73 |  |  |  |  |  |  |
|  | Organization  ~Responsibility |  | -.65 | -.75 |  |  |  |  |  |  |
|  | Productiveness  ~Responsibility |  | -.82 | -.90 |  |  |  |  |  |  |

# Table S6: Correlation coefficients between means of the domains and the facets in the BFI-2-J

|  |  | 1 | 2 | 3 | 4 | 5 | 6 | 7 | 8 | 9 | 10 | 11 | 12 | 13 | 14 | 15 | 16 | 17 | 18 | 19 | 20 |
| --- | --- | --- | --- | --- | --- | --- | --- | --- | --- | --- | --- | --- | --- | --- | --- | --- | --- | --- | --- | --- | --- |
| 1 | Extraversion |  | .16 | .11 | -.18 | .24 | .91 | .84 | .86 | .26 | -.04 | .13 | -.07 | .24 | .13 | -.20 | -.26 | -.01 | .19 | .06 | .35 |
| 2 | Agreeableness | .30 |  | .25 | -.38 | .07 | .16 | .08 | .17 | .76 | .76 | .78 | .13 | .28 | .21 | -.29 | -.30 | -.41 | .01 | .06 | .09 |
| 3 | Conscientiousness | .41 | .45 |  | -.09 | -.01 | .03 | .22 | .04 | .23 | .28 | .08 | .82 | .81 | .83 | .00 | -.01 | -.21 | .08 | -.06 | -.01 |
| 4 | Negative Emotionality | -.47 | -.47 | -.39 |  | .16 | -.20 | -.18 | -.08 | -.12 | -.28 | -.47 | .00 | -.15 | -.07 | .90 | .89 | .83 | .24 | .16 | -.04 |
| 5 | Open-Mindedness | .43 | .35 | .39 | -.26 |  | .17 | .21 | .26 | .17 | -.03 | .02 | .01 | .03 | -.06 | .16 | .13 | .12 | .74 | .83 | .72 |
| 6 | Sociability | .89 | .29 | .28 | -.40 | .30 |  | .64 | .69 | .24 | -.01 | .12 | -.10 | .16 | .04 | -.21 | -.27 | -.05 | .13 | .03 | .26 |
| 7 | Assertiveness | .84 | .26 | .48 | -.44 | .44 | .59 |  | .57 | .18 | -.08 | .07 | .04 | .27 | .24 | -.18 | -.21 | -.07 | .19 | .02 | .34 |
| 8 | Energy Level | .86 | .23 | .31 | -.37 | .38 | .67 | .60 |  | .26 | -.02 | .14 | -.13 | .20 | .07 | -.13 | -.20 | .11 | .18 | .11 | .33 |
| 9 | Compassion | .39 | .84 | .42 | -.30 | .40 | .36 | .34 | .30 |  | .40 | .36 | .14 | .23 | .21 | -.05 | -.09 | -.17 | .16 | .11 | .13 |
| 10 | Respectfulness | .13 | .85 | .45 | -.33 | .25 | .17 | .10 | .07 | .57 |  | .40 | .17 | .25 | .27 | -.20 | -.17 | -.37 | -.09 | .03 | -.04 |
| 11 | Trust | .23 | .78 | .22 | -.54 | .20 | .19 | .20 | .21 | .48 | .50 |  | .01 | .18 | .02 | -.41 | -.42 | -.40 | -.06 | -.01 | .11 |
| 12 | Organization | .20 | .28 | .85 | -.18 | .29 | .09 | .30 | .12 | .26 | .32 | .10 |  | .43 | .52 | .09 | .06 | -.14 | .08 | .01 | -.06 |
| 13 | Productiveness | .45 | .41 | .87 | -.43 | .35 | .32 | .47 | .39 | .36 | .40 | .25 | .58 |  | .56 | -.10 | -.09 | -.21 | .09 | -.07 | .07 |
| 14 | Responsibility | .40 | .47 | .85 | -.40 | .35 | .31 | .46 | .28 | .46 | .45 | .23 | .57 | .62 |  | .00 | .00 | -.17 | .02 | -.09 | -.04 |
| 15 | Anxiety | -.44 | -.29 | -.24 | .90 | -.17 | -.37 | -.41 | -.37 | -.15 | -.16 | -.42 | -.06 | -.30 | -.26 |  | .78 | .58 | .25 | .19 | -.07 |
| 16 | Depression | -.51 | -.39 | -.31 | .91 | -.24 | -.43 | -.44 | -.44 | -.25 | -.25 | -.49 | -.11 | -.36 | -.33 | .81 |  | .57 | .21 | .14 | -.07 |
| 17 | Emotional Volatility | -.28 | -.55 | -.47 | .81 | -.27 | -.25 | -.30 | -.16 | -.39 | -.47 | -.50 | -.29 | -.46 | -.46 | .56 | .58 |  | .15 | .10 | .03 |
| 18 | Intellectual Curiosity | .30 | .20 | .30 | -.08 | .71 | .20 | .34 | .24 | .28 | .16 | .03 | .26 | .25 | .25 | .01 | -.06 | -.16 |  | .43 | .37 |
| 19 | Aesthetic Sensitivity | .18 | .29 | .15 | -.09 | .80 | .12 | .15 | .19 | .28 | .22 | .21 | .11 | .13 | .14 | -.04 | -.09 | -.12 | .34 |  | .35 |
| 20 | Creative Imagination | .54 | .31 | .48 | -.42 | .79 | .38 | .57 | .46 | .37 | .19 | .19 | .32 | .45 | .45 | -.35 | -.40 | -.35 | .47 | .39 |  |
| *Note.* The correlation coefficients of the student sample are presented above the diagonal (*N*=487). The correlation coefficients of the community sample are presented below the diagonal (*N*=500). | | | | | | | | | | | | | | | | | | | | | |

# Table S7: Factor loadings using the mean scores of each of the 15 facets (EFA) in the community sample

|  |  | E | A | C | N | O |
| --- | --- | --- | --- | --- | --- | --- |
| Extraversion | |  |  |  |  |  |
|  | Sociability | **.78** | .16 | .08 | -.18 | .09 |
|  | Assertiveness | **.62** | -.02 | .33 | -.25 | .29 |
|  | Energy Level | **.77** | .04 | .10 | -.19 | .19 |
| Agreeableness | |  |  |  |  |  |
|  | Compassion | .26 | **.66** | .22 | -.03 | .29 |
|  | Respectfulness | -.03 | **.74** | .32 | -.11 | .09 |
|  | Trust | .07 | **.61** | .01 | -.43 | .08 |
| Conscientiousness | |  |  |  |  |  |
|  | Organization | .01 | .10 | **.75** | .00 | .14 |
|  | Productiveness | .27 | .19 | **.70** | -.21 | .12 |
|  | Responsibility | .20 | .27 | **.69** | -.16 | .16 |
| Negative Emotionality | |  |  |  |  |  |
|  | Anxiety | -.25 | -.06 | -.07 | **.87** | .01 |
|  | Depression | -.32 | -.17 | -.10 | **.81** | -.04 |
|  | Emotional Volatility | -.02 | -.39 | -.35 | **.57** | -.10 |
| Open-Mindedness | |  |  |  |  |  |
|  | Intellectual Curiosity | .16 | .06 | .22 | .07 | **.56** |
|  | Aesthetic Sensitivity | .05 | .22 | -.01 | -.02 | **.55** |
|  | Creative Imagination | .32 | .01 | .32 | -.29 | **.67** |
| *Note.* Facet scores were calculated using the average of four items in each facet. Absolute loadings of .50 or stronger are bolded. Cumulative proportion of variance is .63. | | | | | | |


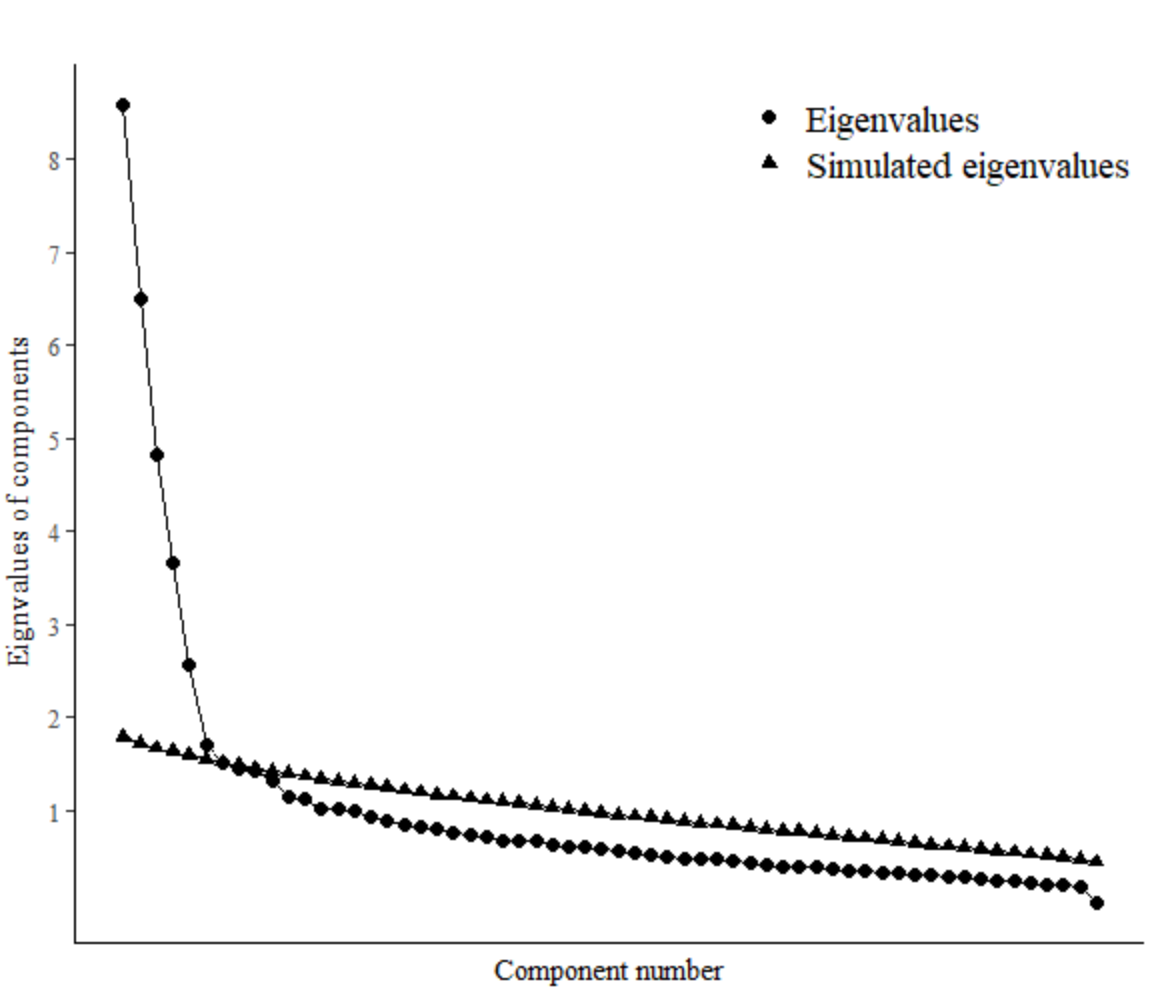


# Figure S1: Eigenvalues from PCA in the student sample


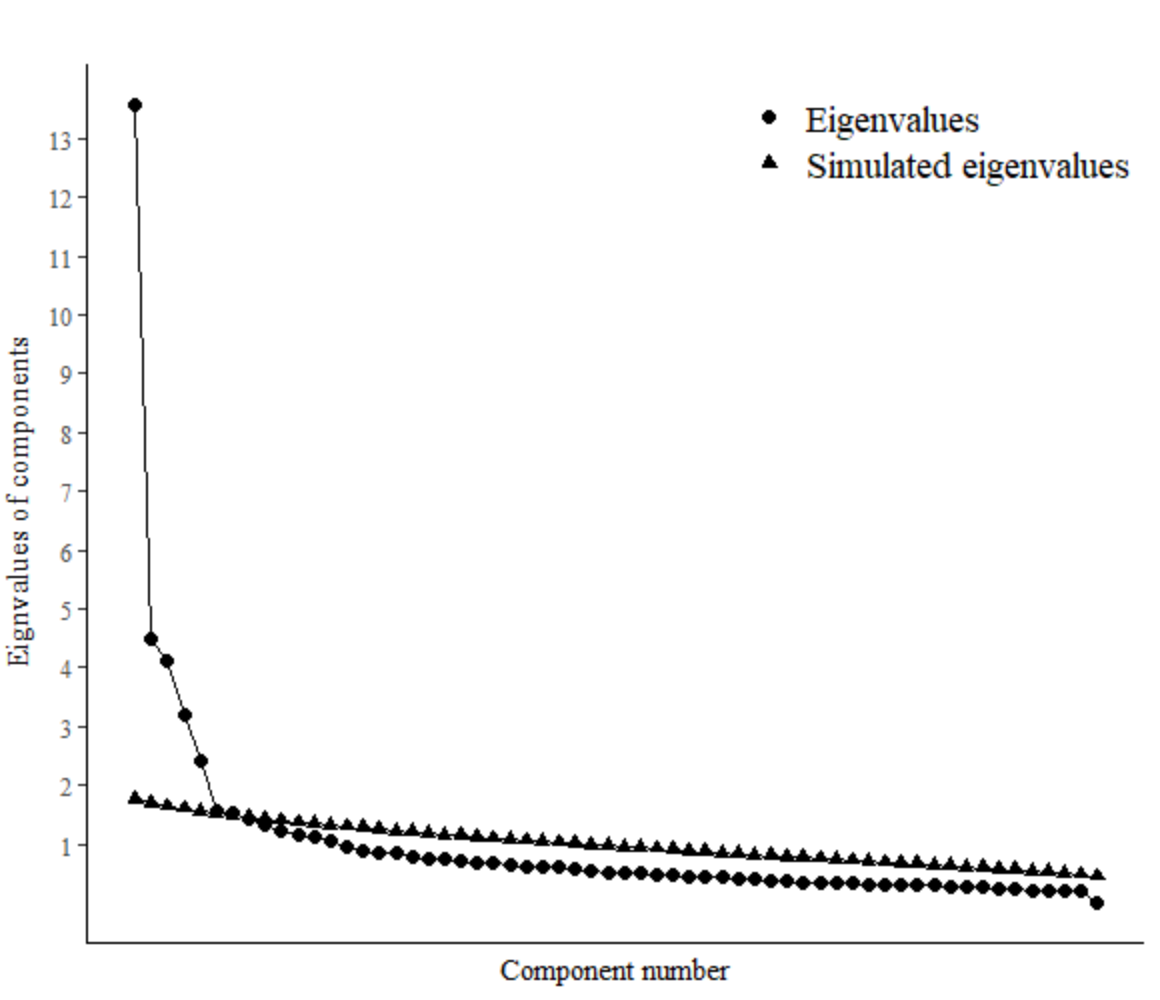


# Figure S2: Eigenvalues from PCA in the community sample
